# Microskeletal stiffness promotes aortic aneurysm by sustaining pathological vascular smooth muscle cell mechanosensation via Piezo1

**DOI:** 10.1038/s41467-021-27874-5

**Published:** 2022-01-26

**Authors:** Weiyi Qian, Tarik Hadi, Michele Silvestro, Xiao Ma, Cristobal F. Rivera, Apratim Bajpai, Rui Li, Zijing Zhang, Hengdong Qu, Rayan Sleiman Tellaoui, Annanina Corsica, Ariadne L. Zias, Karan Garg, Thomas Maldonado, Bhama Ramkhelawon, Weiqiang Chen

**Affiliations:** 1 Department of Mechanical and Aerospace Engineering, New York University, Brooklyn, NY USA; 2 Division of Vascular and Endovascular Surgery, Department of Surgery, New York University Langone Medical Center, New York, NY USA; 3 Department of Biomedical Engineering, New York University, Brooklyn, NY USA; 4 Department of Cell Biology, New York University Langone Medical Center, New York, NY USA

**Keywords:** Biophysical methods, Biophysics, Aneurysm, Biomedical engineering

## Abstract

Mechanical overload of the vascular wall is a pathological hallmark of life-threatening abdominal aortic aneurysms (AAA). However, how this mechanical stress resonates at the unicellular level of vascular smooth muscle cells (VSMC) is undefined. Here we show defective mechano-phenotype signatures of VSMC in AAA measured with ultrasound tweezers-based micromechanical system and single-cell RNA sequencing technique. Theoretical modelling predicts that cytoskeleton alterations fuel cell membrane tension of VSMC, thereby modulating their mechanoallostatic responses which are validated by live micromechanical measurements. Mechanistically, VSMC gradually adopt a mechanically solid-like state by upregulating cytoskeleton crosslinker, α-actinin2, in the presence of AAA-promoting signal, Netrin-1, thereby directly powering the activity of mechanosensory ion channel Piezo1. Inhibition of Piezo1 prevents mice from developing AAA by alleviating pathological vascular remodeling. Our findings demonstrate that deviations of mechanosensation behaviors of VSMC is detrimental for AAA and identifies Piezo1 as a novel culprit of mechanically fatigued aorta in AAA.

## Abstract (web summary)

Mechanobiological signals have been reported to contribute to abdominal aortic aneurysm (AAA) development. Here the authors report that the microskeletal stiffness and a Piezo1-mediated mechanism influence vascular smooth muscle cell mechanosensation and AAA disease development.

## Introduction

Abdominal aortic aneurysm (AAA) is a complex and lethal vascular disease with high incidence worldwide^1,2^. The early stages of the disease onset are characterized by an enlargement of the aortic diameter that implies vascular inflammation and intricate damage to the extracellular matrix (ECM)^3,4^. Despite cumulative research efforts that unmasked pathological signals that contribute to AAA development, the mechanisms underlying AAA progression and the factors that further feed ECM damage are poorly understood. Vascular smooth muscle cells (VSMCs) are key regulators of ECM net composition and their strategic location within the vascular wall suggests that they could serve as spatiotemporal mechanical rheostats. However, how mechanosensation of VSMC is altered within the misshaped silhouette of AAA is ill-defined.

Hereditary mutations of genes controlling VSMC cytoskeleton (CSK) contractile function and mechanosensation have been demonstrated to cause thoracic aortic aneurysm (TAA)^5^. Due to the dynamic nature of the CSK machinery, it plays a critical role in the regulation of mechanoadaptation of cells within their microenvironment^6^. Evidence suggests that abnormal acclimation of VSMC to biomechanical perturbations, such as increased circumferential stress in hypertension can stimulate AAA development^7–9^. However, insights into the change of VSMC CSK integrity and the resulting pathological mechanosensation in AAA have not been explored.

CSK integrity plays a vital role in mediating downstream mechanotransduction responses through mechanosensitive ion channels^10–12^. Piezo1, one of such mechanical sensors, is positioned at the cell membrane of cells and licenses the entry of ions, including Ca^2+^ upon its activation driven by mechanical force^13–17^. Furthermore, Piezo1 channels have been shown to act as critical regulators of the vascular development^18,19^, blood pressure^20^, and hypertension-dependent arterial remodeling^21^. However, the behavior of Piezo1 channel in VSMC within a mechanically compromised AAA microenvironment is undefined. In addition to the mechanical stimuli, molecular signals concentrate within the AAA sac and might impact mechanotransduction signals in VSMC. We have previously shown that neuronal guidance proteins can intervene in aortic aneurysms^22^. Notably, we demonstrated that Netrin-1 was released from transmural macrophages and promoted AAA by sustaining downstream Ca^2+^ signals necessary to stimulate matrix degrading metalloproteinase MMP3 in VSMC^23^. This suggested that the pathological proteolytic switch of VSMC triggered by Netrin-1 in AAA could be driven by microskeletal alterations via Piezo1.

Here, we show that the transition of VSMC to a mechanically stiffer state during AAA development is driven by elevated CSK crosslinking protein, α–actinin2. Loss-of-function of Netrin-1 prevented AAA development as previously shown^23^ and relieved arterial tension by inhibiting α–actinin2 expression. Using a sophisticated ultrasound tweezers-based micromechanical system, we experimentally and theoretically captured dynamic stiffness and impaired mechanosensing profiles of VSMC niched in AAA. Stiffened VSMC membrane powered mechanical energy necessary to induce and activate mechanoresponsive Ca^2+^ channel Piezo1. Antagonizing Piezo1 refrained matrix degradation and prevented the development of AAA. Our study reveals a molecular connection between defective mechanosensation and pathological vascular remodeling and attribute the overactivation of Piezo1 as a critical mediator of these effects.

## Results

### CSK crosslinking is increased in VSMC in stiffened aortic tissue in AAA

**Fig. 1:**
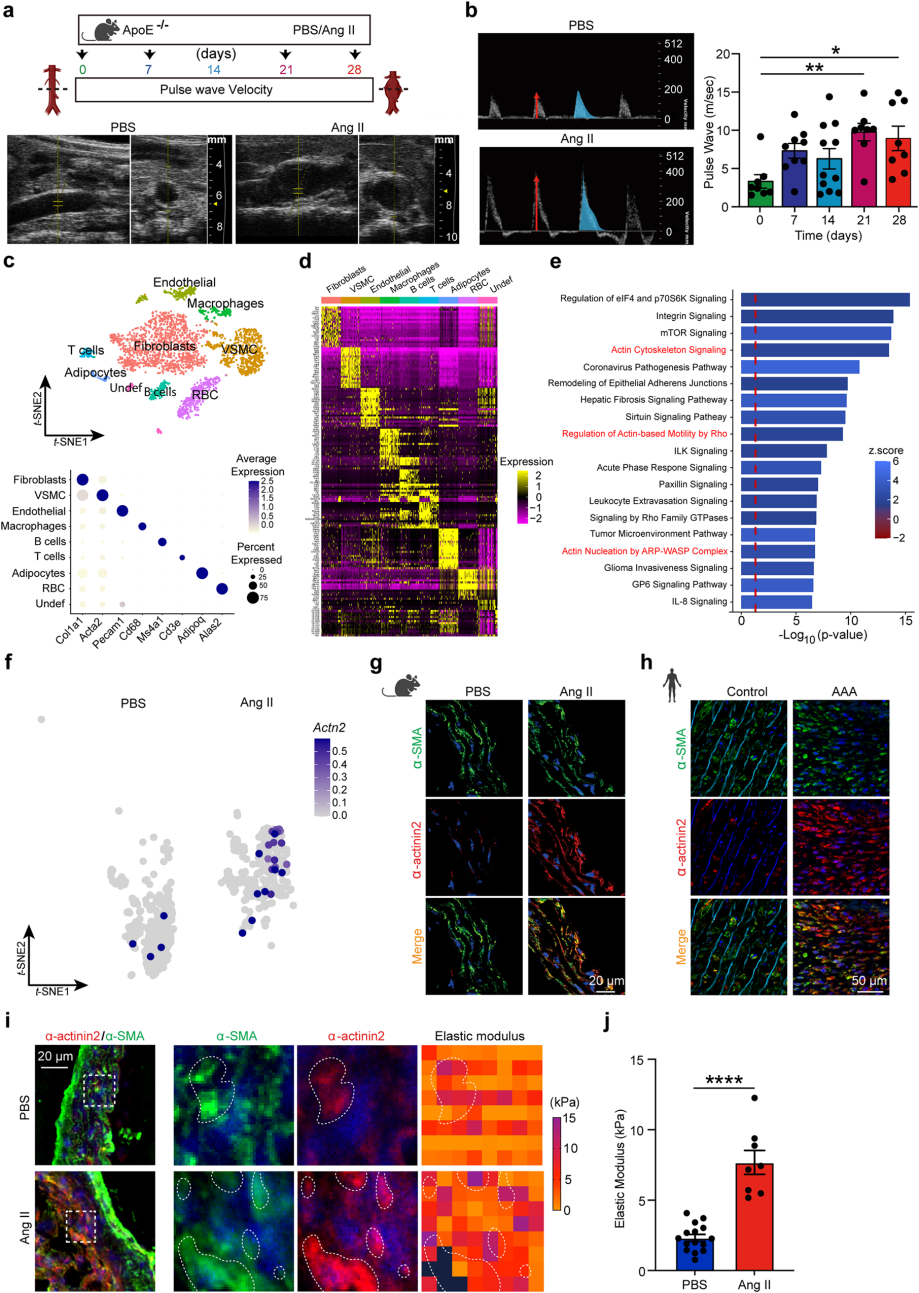
Increased CSK crosslinking in VSMC of the stiffened aortic tissue in AAA. **a** Schematic representation of experimental protocol of PBS (control) or Angiotensin II (Ang II) in mice with timeline of pulse wave velocity (PWV) measurements. **b** Representative images of ultrasonography 2D scan images of peak systolic velocity (red arrow), global pulse wave curve (blue curve), and PWV quantification of mice exposed to Ang II for days as indicated. *n* = 8 mice for day 0, *n* = 9 for day 7, *n* = 11 for day 14, *n* = 8 for day 21, and *n* = 8 for day 28. One-way ANOVA followed by Dunnett’s multiple comparisons test. *P = 0.0136 and **P = 0.0041. Data are presented as mean values ± SEM. **c** *t*-distributed stochastic neighbor embedding (*t*-SNE) plot of single-cell RNA-sequencing of mice aortas (*n* = 4–5 per group). Cell clusters are annotated, top panel. Dot plot representation showing average expression and percentage of expression in the cluster of the markers used for cluster identification, lower panel. VSMC vascular smooth muscle cells, RBC red blood cells. **d** Heatmap of top differentially expressed transcripts of each cell clusters. **e** Pathway analysis of differentially expressed genes in AngII vs PBS VSMC. **f** *t*-SNE distribution of *Actn2* mRNA (log_2_ expression) within the VSMC cluster and across conditions, as indicated. **g**, **h** Immunofluorescence (IF) staining of α-actinin2 (red) and α-SMA (green) in murine (**g**) and human (**h**). **i** Representative IF merged image of α-SMA (green) and α-actinin2 (red) in aortic section of mice (left panel, dashed box shows area of atomic force microscopy (AFM) measurement) and heatmap representation of elastic modulus captured by AFM (right panel, dotted lines demark regions enriched in α-actinin2 with maximum force). Nuclei are stained with DAPI (blue) in all IF images. **j** Quantification of the elastic modulus of aortic sections as indicated. *n* = 15 square regions in at least three aortic sections for PBS and *n* = 8 square regions in at least three aortic sections for Ang II. Two-sided unpaired *t*-test. ****P < 0.0001. Data are presented as mean values ± SEM. Tissue sections in **g**, **h**, and **i** were transversal sections. Mouse, human, and aorta icons in **a**, **g**, and **h** are created with BioRender.com. Source data are provided as a Source Data file.

To determine the mechanical properties of VSMC in AAA, we measured the level of stiffness in the vascular wall in experimental AAA. We first compared aortic stiffness of diseased and control ApoE^−/−^ murine aortas (Fig. 1a) captured by pulse wave velocity (PWV) weekly using the diameter-velocity method^24^. Aortic PWV revealed that the vessel wall stiffness was incrementally increased in AAA compared to controls (Fig. 1b), consistent with a previous report^25^. An increase in both peak velocity value (Fig. 1b, red arrow) and amplitude of the wave (Fig. 1b, blue curve) were observed in the AAA sac compared to that from an equivalent pararenal region of non-diseased aortas. These results demonstrate that local stiffness is amplified in the aneuvrysmal sac.

To delve into the molecular mechanisms underlying the accrual of tension observed in AAA, we compared mechanosensitive signatures of healthy and angiotensin II (Ang II)-induced aneurysmal abdominal aortas by single-cell RNA-sequencing (Fig. 1c). Using the principal component analysis (PCA) nonlinear dimension-reduction method, nine distinct aortic sub-populations were identified as per their genetic distribution patterns as shown in the *t*-distributed stochastic neighbor embedding (*t*-SNE, Fig. 1c). These results were consistent with the transcriptome signature of an independent set of published single-cell RNA sequencing of AAA modeled by elastase infusion^26^ (Supplementary Fig. 1a–c). We focused on the analysis of VSMC clusters based on our previous findings demonstrating reprograming of VSMC by Netrin-1^23^. VSMC were determined based on expression level of markers including α-actin2 (*Acta2*) (Fig. 1c, bottom panel and Supplementary Fig. 1c), Myosin heavy chain 11 (*Myh11*) and Myosin regulatory light chain 9 (*Myl9*) (Fig. 1d and Supplementary Fig. 1c). A set of unbiased genes discriminated the identity of each cell type as represented by the heatmap (Fig. 1d). Utilizing an adjusted nominal *P* value of ≤0.05, we captured a large range of transcriptome with differential expression patterns in VSMC niched in AAA compared to controls (Supplementary data. 1). Enrichment analysis using Ingenuity Pathway Analysis (IPA) software revealed that several signaling pathways were altered in VSMC from AAA. Notably, Several genetic networks involved in the regulation of actin CSK were enriched in VSMC from AAA (Fig. 1e). Based on these results we hypothesized that α-actinin2 (Actn2), a key regulator of actin filaments crosslinking^27,28^ could be enriched in aneurysmal VSMC. Indeed the elevated expression of *Actn2* mRNA (Fig. 1f) and protein were elevated in VSMC as detected by immunofluorescence staining of aortic sections of Ang II- (Fig. 1g) and elastase-induced AAA (Supplementary Fig. 1e). Notably, analysis of images captured from human AAA and control sections demonstrated a similar pattern of expression characterized by an increased level of α-actinin2 within VSMC in AAA (Fig. 1h). Atomic force microscopy (AFM) nanoindentation assays revealed a higher distribution of intra-aortic force magnitudes in aneurysmal sections compared to non-diseased sections which peaked in nanoscale regions enriched in α-actinin2 indicative of stiffened territories within the tissue (Fig. 1i). Quantification of mean elastic modulus revealed elevated stiffness in aortic tissues analyzed from aneurysmal animals compared to control tissues (Fig. 1j). This pattern was maintained throughout different regions of interest captured at cardinal points around the perimeter of the aortic ring and the level of α-actinin2 expression correlated positively with the elastic modulus of the aortic tissue (Supplementary Fig. 2a–i). These results suggested that α-actinin2 is a major integrator of stiffness of VSMC thereby reinforcing mechanical strength in the vascular wall during AAA development.

### Micromechanical system maps defective mechanosensation of VSMC in AAA

**Fig. 2:**
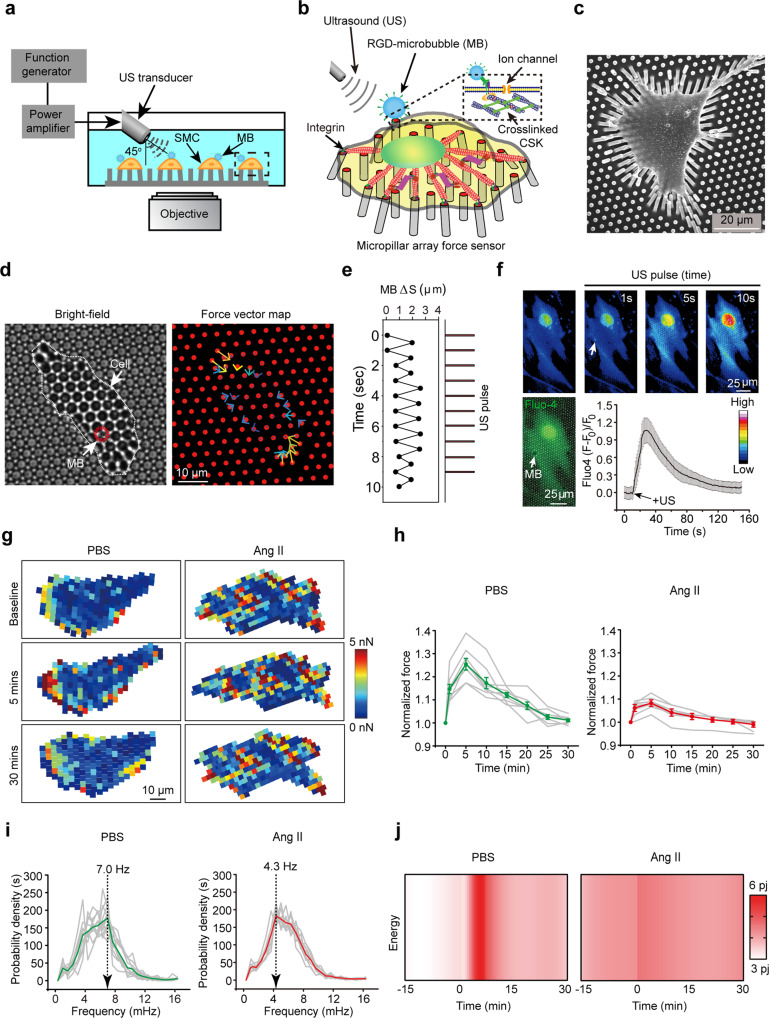
Defective mechanosensation of VSMC in AAA. **a** A schematic showing the integrated micromechanical system by using ultrasound (US) excitation of microbubbles (MB) attached via RGD-integrin binding to the membrane of VSMC seeded on the PDMS micropillar array. **b** Diagram illustrating micropillar-based mechanical force sensor and ultrasound tweezer system in single cell. **c** Representative scanning electron microscopy (SEM) image of single VSMC seeded on PDMS micropillars. **d** Representative microscopy images of bright field (left panel) and red channel showing micropillars (right panel). VSMC is delineated by white dotted line, microbubble (MB) is delineated by red dotted circle blue and yellow arrows represent force vector. **e** Relative microbubble movement in response to 10-s, 1 Hz transient ultrasound stimulation. **f** Representative microscopy images of green channel showing Fluo-4 Ca^2+^ probe and quantification of Ca^2+^ influx in response to US stimulation. *n* *=* 13 cells. Data are presented as mean ± SEM. **g** Representative heatmap representation of baseline micropillar traction force or in response to US stimulation (5 and 30 min) of single VSMC isolated from PBS or Ang II treated mice. **h**, **i** Normalized global traction force (**h**) and mean frequency (**i**) of VSMC response to ultrasound stimulation. The frequency responses in **i** represent VSMC force dynamics from 0 to 5 min. In **h**, data are presented as mean ± SEM (green and red curves), individual values are shown in gray. *n* = 8 cells per group. In **i**, data are presented as mean (green and red curves) and individual values are shown in gray. *n* = 12 cells for PBS and *n* = 9 cells for Ang II. *P* value was calculated using Kolmogorov–Smirnov (KS) test. *P* = 0.010364. **j** Representative temporal heatmap of consumed energy in VSMC isolated from PBS or Ang II treated mice in response to ultrasound stimulation. Source data are provided as a Source Data file.

To evaluate whether the level of mechanosensation of a single VSMC niched in non-diseased aortic wall undergoes a pathological switch in aneurysmal milieu, we engineered a sophisticated single-cell micromechanical system (Fig. 2a, b) consisting of ultrasound tweezers and an elastic micropillar-based mechanical sensor allowing measurements of baseline CSK tension at the cell membrane as well as the response to ultrasound stimulus mediated by a microtubule system^29–31^. An electron microscopy captured image of VSMC adhering to a polydimethylsiloxane (PDMS) micropillar substrate (Fig. 2c) revealed displacement of micropillars at the circumference of VSMC. This coincided with images of clustered micropillars at the periphery regions of VSMC with larger force (Supplementary Fig. 3a–d). For the tweezers, we applied a 10-s, 1 Hz, ~100 pN transient and local force to a single VSMC through an RGD-integrin bonded lipid-encapsulated microbubble on the cell membrane under ultrasound excitation (Fig. 2d). Activation of the system by ultrasound pulses generated acoustic radiation force on the microbubbles, causing its displacement (Fig. 2e and Supplementary Fig. 3e) and therefore, allowing the application of a controllable mechanical stress on cells through a microbubble-integrin-actin CSK interaction as shown in the inset in Fig. 2b. We first tested whether ultrasound-induced transient mechanical stimulation could activate Ca^2+^ ion influx in VSMC isolated from mouse abdominal aortas. Immediately after the application of ultrasound stimulation, Ca^2+^ influx, assessed by fluo-4 calcium sensor probe, was clearly detected in VSMC (Fig. 2f), suggesting that the microbubble-integrin-actin CSK linkage can serve as a mechanosensory signal to trigger intracellular mechanical transduction.

We then applied our integrated single-cell micromechanical system to capture mechanosensation response of primary VSMC isolated from control or aneurysmal microenvironments. Within 30 mins after the onset of the 10-s ultrasound stimulation, both healthy and AAA VSMC CSK tension exhibited a biphasic dynamic, in which the single-cell level CSK tension increased continuously within the reinforcement period (0–5 min) and restored to their ground state in the relaxation period (5–30 min) as depicted by force map of individual cells and quantification of normalized force over 30 min (Fig. 2g). No significant change was observed in unstimulated cells (Supplementary Fig. 3f). In-depth characterization of temporal CSK tension during instantaneous mechanosensation revealed that while VSMC from AAA milieu generally have a higher CSK tension at the ground state, they exhibited a compromised ability to generate mechanoallostatic force compared to control (Fig. 2h).

To further delve into the intrinsic alterations of CSK associated with the differential mechanosensation of VSMC in AAA, we performed an instantaneous frequency spectrum analysis, due to its profound sensitivity in capturing intrinsic CSK characteristics and mechanosensation based on Hilbert–Huang transform (HHT)^32^. Decomposition of the subcellular CSK tension by HHT (Supplementary Fig. 4a) generated distinct intrinsic mode functions (IMFs) at a unicellular level of VSMC before and after ultrasound stimulus (Supplementary Fig. 4b, c). We observed intrinsic blue shift of VSMC CSK tension instantaneous frequency spectra after ultrasound stimulation (Supplementary Fig. 4d), indicating that the CSK tension frequency spectrum encodes the wiring of the molecular circuitry that regulates VSMC mechanosensation. When comparing the instantaneous frequency spectra for healthy and AAA VSMC, we found that VSMC from AAA displayed an intrinsic blunt in mechanosensitivity as demonstrated by a significant decrease in response frequency (Fig. 2i and Supplementary Fig. 4e). Such changes of VSMC mechanosensation were further featured by generating a combined frequency-amplitude energy heatmap to reflect the decrease of energy consumed by VSMC in pathological mechanosensation in AAA (Fig. 2j). Taken together, we demonstrate that single-cell mechanosensation characterization is a reliable technique capable of capturing CSK-mediated mechanosensitive imprints of VSMC niched in AAA milieu.

### VSMC microskeletal force and mechanosensation are temporally impaired in AAA

**Fig. 3:**
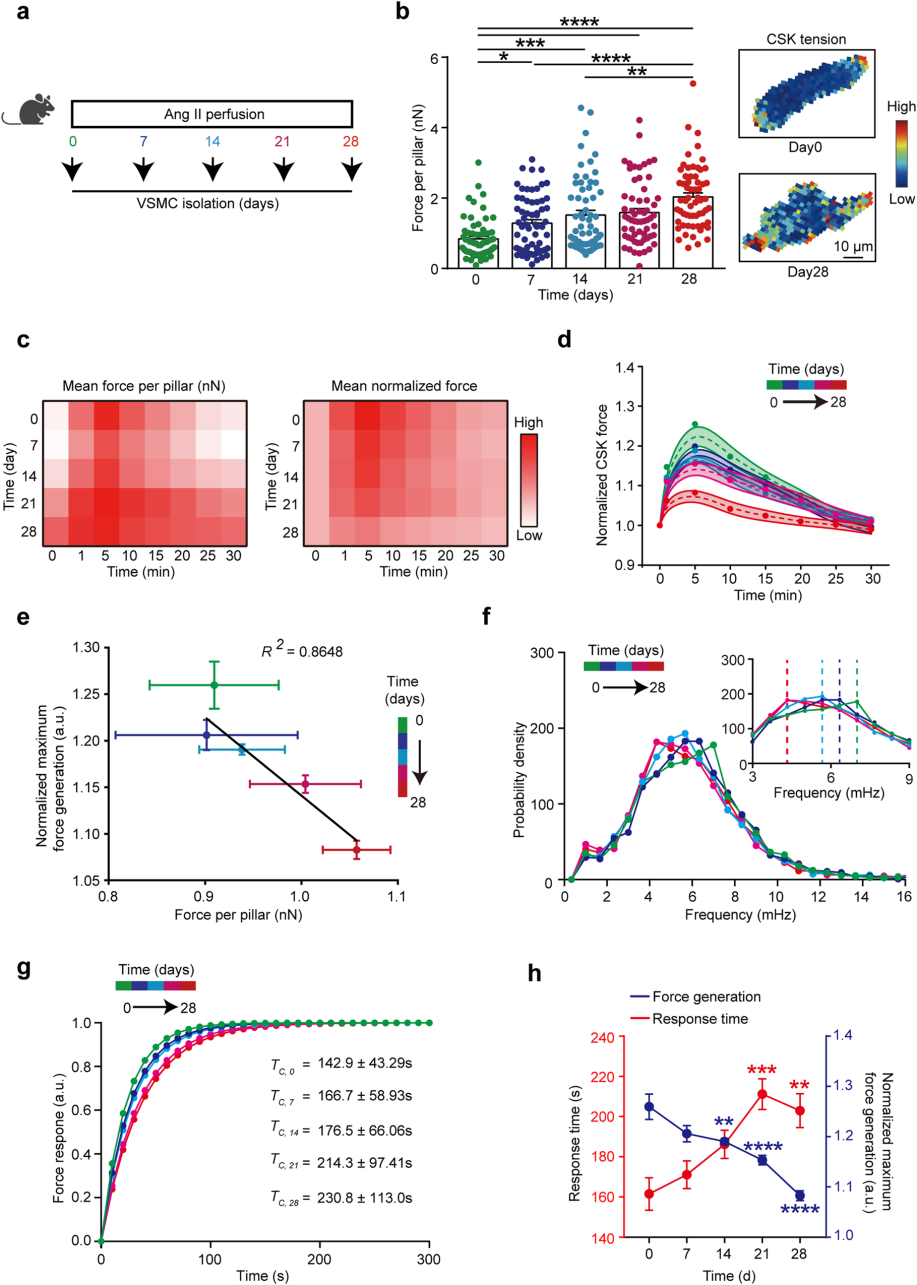
Temporal evolution of VSMC mechanosensation during AAA. **a** Schematic of experimental protocol for VSMC isolation. **b** Quantification and representative heatmaps (day 0 and 28) of mean basal tension per pillar in VSMC extracted from aorta at timepoint indicated. *n* = 63 cells for day 0, *n* = 63 for day 7, *n* = 62 for day 14, *n* = 61 for day 21, and *n* = 61 for day 28. Kruskal–Wallis test followed by Dunn’s multiple comparisons test. *P = 0.0151, **P = 0.0043, ***P = 0.0002, ****P < 0.0001. Data are presented as mean values ± SEM. **c** Heat maps of temporal evolution of mean force per pillar (left) and mean normalized tension (right) of VSMC over time during instantaneous mechanosensation. **d** Temporal profiles for each time point (color coded as in **a**) of normalized CSK tension per pillar of VSMC in response to 10-s mechanical stress. *n* = 8 cell per group, data are presented as mean value ± SEM. **e** Correlation analysis of VSMC progressive basal CSK tension in AAA and maximum force generation in instantaneous mechanosensation. *n* = 8 cells per group. Data are presented as mean values ± SEM. **f**, **g** Instantaneous frequency distribution (**f**) and characteristic curves for deriving response time (**g**) of VSMC instantaneous mechanosensation. In **f** and **g**, each curve represents the mean value. Kolmogorov–Smirnov (KS) test was performed to determine the significant decrease of frequency distribution and force response between VSMC extracted from control aorta and aorta exposed to Ang II for 28 days, *P* = 0.010364. **h** Force generation ability (blue) and response time (red) of VSMC extracted from aorta exposed to Ang II at time points as indicated by color code. For force generation, *n* = 8 cells per group. One-way ANOVA followed by Dunnett’s multiple comparisons test. **P = 0.0086, ****P < 0.0001; For response time, *n* = 12 cells for day 0, *n* = 6 for day 7, *n* = 8 for day 14, *n* = 10 for day 21, and *n* = 9 for day 28. Kruskal–Wallis test followed by Dunn’s multiple comparisons test. **P = 0.0049, ***P = 0.0004. Data are presented as mean values ± SEM. a.u., arbitrary unit. Mouse icon in **a** is created with BioRender.com. Source data are provided as a Source Data file.

We next delineated the time point during AAA disease progression, modeled by continuous angiotensin II (Ang II) treatment in mice, at which VSMC endure a shift in CSK mechanosensation. VSMC were isolated from the abdominal aorta following 7, 14, 21, and 28 days of Ang II infusion (Fig. 3a) which allowed the study of mechanosensation behaviors of VSMC from early, mid and advanced stages of AAA development. We observed a progressive increase of ground state CSK tension (Fig. 3b) in VSMC over the experimental course of AAA development. The observed increase of VSMC CSK tension in AAA development is independent of the substrate stiffness and is accompanied by the increase of α-actinin2 expression (Supplementary Fig. 5a–c). Interestingly, instantaneous mechanosensation characterized by the dynamic force generation of VSMC showed an opposite trend compared to their mechanobiological ground states (Fig. 3c, d). By mapping the VSMC mechanobiological responses, we discovered a negative correlation between VSMC instantaneous mechanosensation and AAA (Fig. 3e). Specifically, VSMC isolated from mice with advanced AAA displayed a significant inability of generating contractile force within the reinforcement period (0–5 min) upon stimulation with a localized stress. By implementing the spatiotemporal sampling and frequency spectrum analysis of global and local CSK tension during mechanosensation, we revealed significant red shift in the frequency spectra of VSMC instantaneous contraction during AAA development (Fig. 3f). Deriving response time as a measure of cell’s sensitivity to mechanical stimulation from the reciprocal of IMF central frequency^33^ in instantaneous mechanosensation further indicated a prolonged response of VSMC mechanosensation as AAA progress (Fig. 3g). Accordingly, response time was increased while maximum force generation was decreased following stimulation (Fig. 3h). Thus, our results indicate that while the basal force of VSMC is increased in AAA, their ability to resonate to mechanical stimuli and generate efficient mechanosensitivity is mitigated.

### Defective CSK-mediated mechanosensation of VSMC can be predicted in AAA

**Fig. 4:**
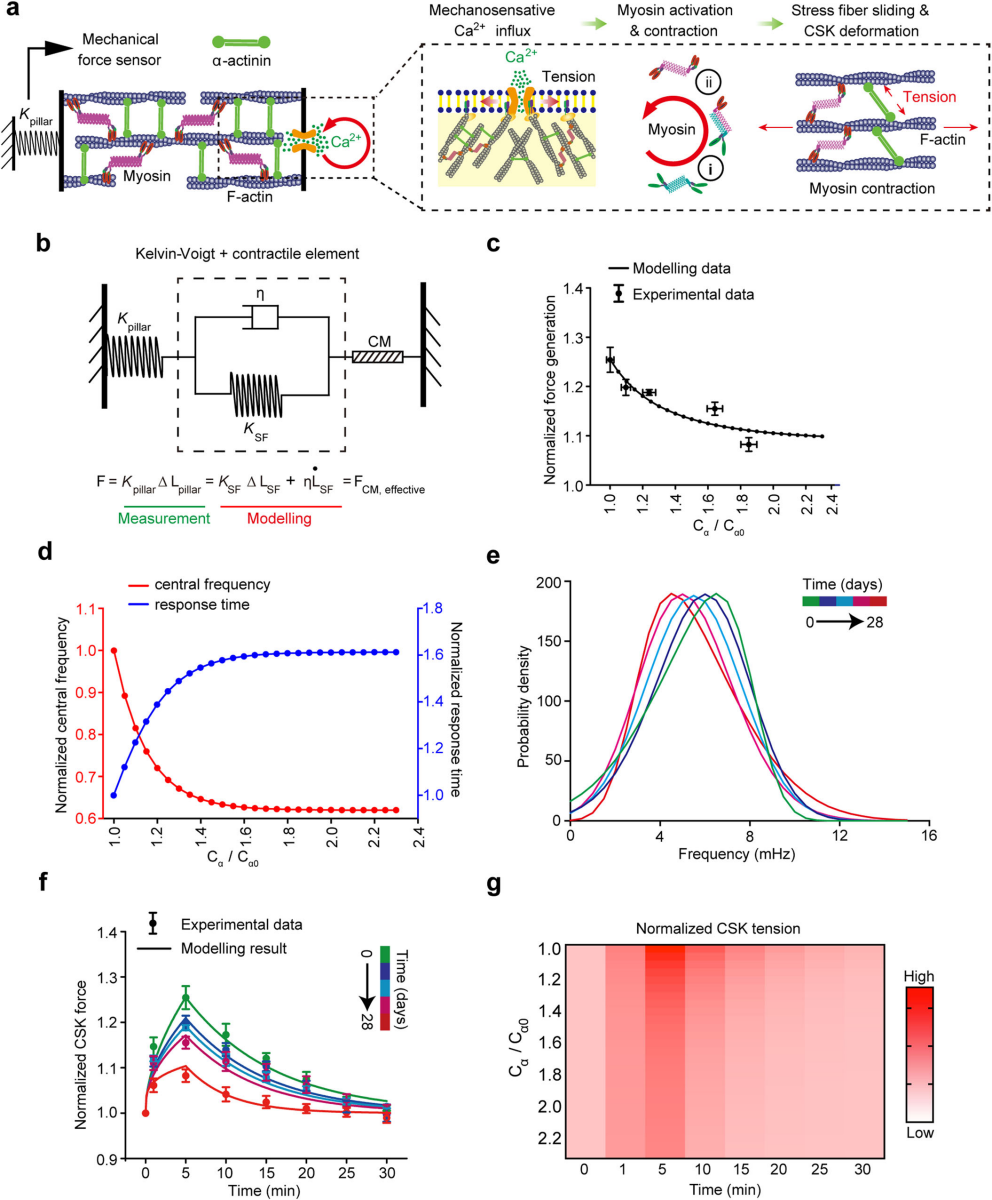
Theoretical modeling of VSMC defective mechanosensation in AAA. **a** Illustration of the theoretical modeling of VSMC mechanosensation involving three key mechanisms: mechanosensitive Ca^2+^ flux, two-step myosin motor activation and contraction, and stress fiber sliding and CSK deformation. **b** Schematic of VSMC mechanosenstion modeled by Kelvin-Voigt element, with a viscoelastic material consisting of elastic stress fiber (SF) with an elastic modulus of ${K}_{{{{{{{\mathrm{SF}}}}}}}}$ and a viscous component with viscosity of $\eta$, connected in series to the contractile myosin element (CM). **c** Force generation ability for VSMC with different expression of α-actinin2 (C_α_/C_α0_) in response to ultrasound tweezer stimulus. Experimental data of maximum force generation in **c** is adapted from Fig. 3h. Experimental data of (C_α_/C_α0_) in **c** is adapted from Supplementary Fig. 5a. **d** Predicted response time (**d**, blue) and normalized instantaneous central frequency (**d**, red) of VMSC mechanosensation with the increase of α-actinin2 (C_α_/C_α0_). **e** Theoretical prediction of instantaneous frequency distributions of VSMC instataneous mechanosensation. **f** Alignment of theoretical prediction (solid curves) and experimental measurements (dots with error bar) of force generation dynamics of VSMC instantaneous mechanosensation. Experimental data of force generation in **f** is adapted from Fig. 3d. **g** Summary heat map of the predicted force generation dynamics of VSMC with different expressions of α-actinin2. Source data are provided as a Source Data file.

In light of our experimental findings, we developed a predictive biophysical kinetics model that incorporated three key actomyosin CSK events: CSK viscoelastic deformation and relaxation, actomyosin activation and contraction, and mechanical-induced Ca^2+^ influx (Fig. 4a). We adopted the Kelvin–Voigt–Myosin (KVM) model^34^ to specifically study how pathological increases of α-actinin2 in VSMC can lead to different mechanosensation dynamics (Fig. 4b) in AAA. In this model, Ca^2+^ influx caused myosin activation and sliding leading to CSK deformation which relied on viscoelasitic properties regulated by α-actinin2 crosslinking. Using this model (Fig. 4a, b; Materials and methods section), we predicted that VSMC mechanosensitivity, as defined by the ability to generate instantaneous force and the response time to mechanical stimulus, are directly regulated by the viscoelasticity properties of the CSK (Fig. 4c, d). Our theoretical model thus captured similar VSMC instantaneous mechanosensation shift (Fig. 4e) as our experimental results associating increased α-actinin2 and red shift in CSK tension frequency spectrum in AAA (Fig. 3f). By directly adapting parameters from the mechanobiological ground states of VSMC in AAA and previous experimental studies (Supplementary Table. 1), our analytical results qualitatively predicted CSK actomyosin dynamics regarding instantaneous CSK tension frequency spectrum (Fig. 4e) and force generation (Fig. 4f, g) in response to mechanical stimulation. Successful simulation of the instantaneous mechanosensation using the biophysical model underscored the critical role of α-actinin2 in regulating VSMC viscoelastic properties.

### Netrin-1 regulates VSMC stiffness and mechanosensation via α-actinin2

**Fig. 5:**
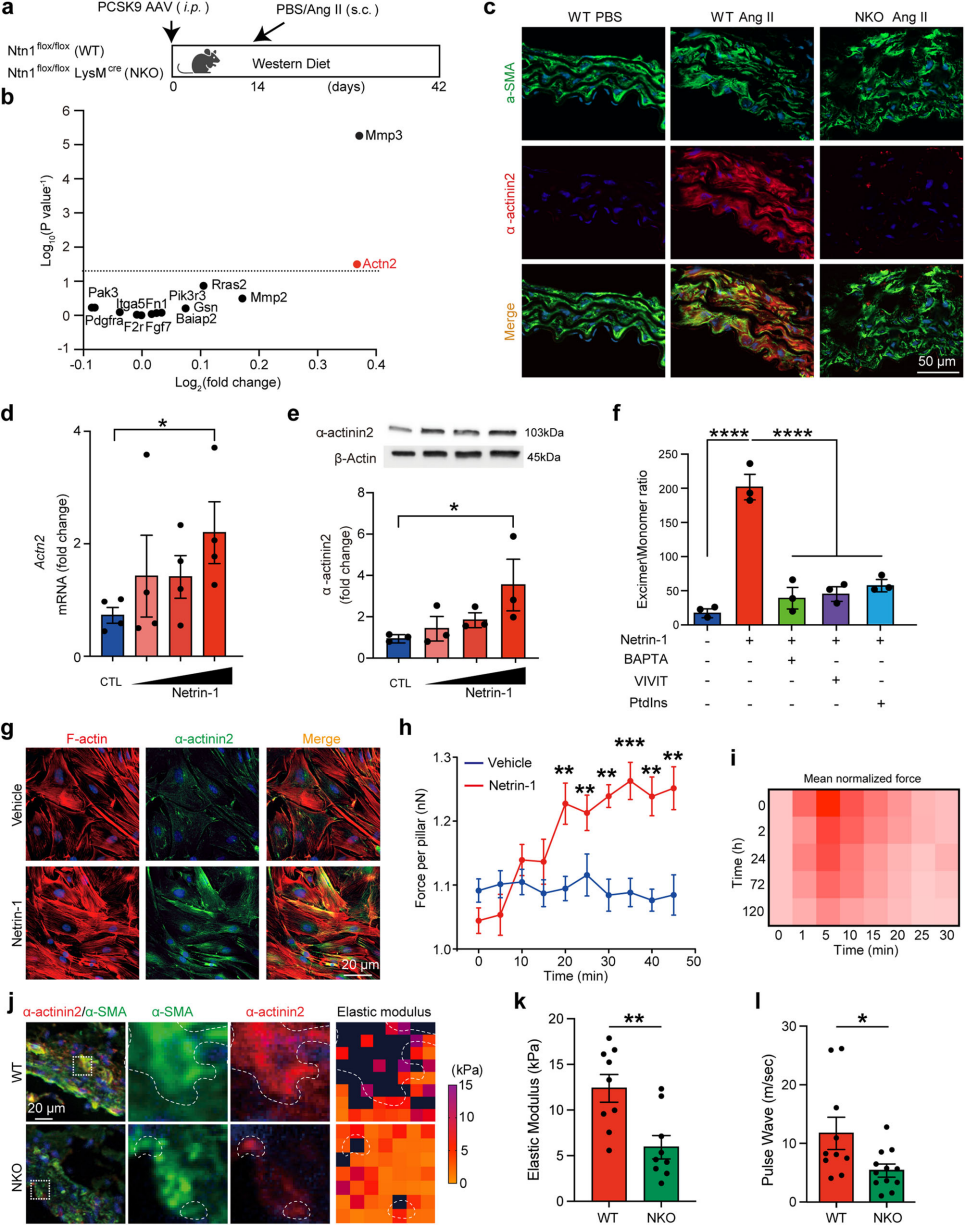
Netrin-1 regulates VSMC CSK crosslinking and mechanosensation via α-actinin2. **a** Schematic representation of AAA in vivo protocol. **b** Dot plot representation of differentially expressed mRNAs associated with CSK regulation in control (WT) or conditional deficiency of Netrin-1 (NKO) aorta. *n* = 4 per group. **c** Immunofluorescence (IF) staining of α-actinin2 (red) and α-SMA (green) in aortic sections of WT and NKO mice treated as indicated. **d** Quantitative- RT-PCR (qPCR) analysis of *Actn2* mRNA. *n* = 4 independent experiments per group. One-way ANOVA followed by uncorrected Fisher’s LSD test. *P = 0.034. Data are represented as mean values ± SEM. **e** Immunoblot of α-actinin2 and β-actin expression in VSMC stimulated with recombinant Netrin-1 (0.625, 1.25, or 2.5 µg/ml) and quantification of bands. *n* = 3 independent experiments per gruop. One-way ANOVA followed by Dunnett’s multiple comparisons test. *P = 0.0383. Data are represented as mean values ± SEM. **f** Membrane fluidity of VSMC treated with Netrin-1 in conditions as indicated. *n* = 3 independent experiments per group. One-way ANOVA followed by Turkey’s multiple comparisons test. ****P < 0.0001. Data are represented as mean values ± SEM. **g** Representative IF images of α-actinin2 (green) and F-actin (red) in VSMC stimulated with Netrin-1 for 24 h. *n* = 10 per group. **h** Force per pillar measurements at indicated time of VSMC stimulated with Netrin-1 or with PBS. *n* = 9 cells for PBS and *n* = 7 for Netrin-1 from three independent experiments. One-way ANOVA followed by Turkey’s multiple comparisons test. **P = 0.0027 and 0.0033 for *t* = 20 and 25 mins respectively; ***P = 0.0006, 0.0007, and 0.0002 for *t* = 30, 40, and 45 mins, respectively; ****P < 0.0001 for *t* = 35 mins. Data are represented as mean values ± SEM. **i** Heatmap profiles of force generation in instantaneous mechanosensation of VSMC stimulated with Netrin-1 for the indicated time. **j** IF staining of α**-**SMA (green) and α-actinin2 (red) of aortic sections from WT or NKO mice treated with Ang II. Dashed boxes show magnified areas of staining and of AFM depicted by heatmap of elastic modulus magnitudes (dotted lines show regions of enriched staining and force). **k** Quantification of elastic modulus of WT and NKO aortas isolated from mice perfused with Ang II. *n* = 9 square regions in at least 3 aortic sections for both WT and NKO. Two-sided unpaired *t*-test. **P = 0.0038. Data are represented as mean values ± SEM. **l** Aortic pulse wave velocity quantification of WT or NKO mice perfused with Ang II. *n* = 10 mice for WT and *n* = 12 for NKO. Two-sided Mann–Whitney test. *P = 0.03. Data are represented as mean values ± SEM. Nuclei are stained with DAPI (blue) in all IF images. Tissue sections in **c** and **j** were transversal sections. Mouse icon in **a** is created with BioRender.com. Source data are provided as a Source Data file.

Since our model accurately predicted that increased CSK stiffness driven by α-actinin2 is critical to evaluate VSMC mechanosensitivity in AAA, we further investigated the upstream mechanisms triggering the induction of α-actinin2 in VSMC. We have previously demonstrated that the conditional deletion of Netrin-1 in monocyte and macrophages Ntn1^flox/flox^LysMcre^+/−^ (NKO) protected mice from developing AAA compared to Ntn1^flox/flox^ mice (WT) when challenged to AAA via Proprotein convertase subtilisin/kexin type 9 serine protease (PCSK9) overexpression combined with Western diet and Ang II infusion^23^ (Fig. 5a). Analysis of published RNA sequencing (RNAseq) dataset of aorta isolated from WT and NKO mice revealed a library of differentially expressed genes (Supplementary data. 2), including the gene encoding *Actn2* (Fig. 5b). This suggested that Netrin-1 could directly regulate the transcription of *Actn2* mRNA in the aortic tissue. Immunofluorescence staining of WT aortic sections demonstrated an elevated presence of α-actinin2 in VSMC from WT mice treated with Ang II compared to WT mice treated with PBS (Fig. 5c). Analysis of sections from NKO mice treated with Ang II that did not develop AAA, revealed a reduction of α-actinin2 in medial VSMC. These results instructed us that Netrin-1 could regulate α-actinin2 both transcriptionally and translationally in VSMC.

To directly demonstrate whether Netrin-1 could regulate α-actinin2 in VSMC, we performed qRT-PCR and western blot analysis which demonstrated that treatment of VSMC with recombinant Netrin-1 dose-dependently increased the expression of *Actn2* transcript and α-actinin2 protein (Fig. 5d, e). To gain further insights into the mechanism of how Netrin-1 could regulate stiffness, we assessed VSMC membrane stiffness in the presence of Ca^2+^ chelator, 1,2-bis(o-aminophenoxy) ethane-N,N,N′,Nʹ-tetraacetic acid (BAPTA), or Ca^2+^-sensitive nuclear factor of activated T-cells cytoplasmic 3 (NFATc3) inhibitor, MAGPHPVIVITGPHEE (VIVIT) peptide, based on our previous data demonstrating that Netrin-1 could activate NFATc3 via Ca^2+^ ^23^. VSMC stiffness was induced by Netrin-1 and mitigated in the presence of BAPTA, VIVIT or α-actinin2 inhibitor, phosphatidylinositol (3,4,5)-trisphosphate (PtdIns(3,4,5)P3) (Fig. 5f). Altogether, these results suggested that AAA-promoting signals such as Netrin-1, could regulate VSMC rigidity by stimulating actin fiber crosslinking via α-actinin2.

To test whether aberrant cytoskeletal stress observed in VSMC isolated from AAA could be recapitulated by sustained exposure of recombinant Netrin-1 in vitro, we monitored biomechanical and mechanosensation responses of VSMC exposed to Netrin-1 for 1 day. Analysis of images captured by fluorescent microscopy revealed an elevated presence of F-actin stress fibers coincident with α-actinin2 in VSMC exposed to Netrin-1 (Fig. 5g). Accordingly, exposure of VSMC to recombinant Netrin-1 increased CSK tension of VSMC captured by micropillar assay (Fig. 5h) corollary to VSMC isolated from AAA milieu suggesting that these changes are intrinsic to AAA disease (Supplementary Fig. 5d). Instantaneous mechanosensation analyses of VSMC treated with recombinant Netrin-1 for different days showed a time-dependent increase of CSK tension and decrease of force generation (Fig. 5i), similar to the pattern we observed with VSMC isolated from AAA over the course of disease development. In-depth stiffness analysis by AFM demonstrated increased expression of α-actinin2 coincident with peaked stiffness in aortic segments of WT mice that developed AAA. In contrast, the overall stiffness of tissue and α-actinin2 expression were reduced in smooth muscle cells harbored in NKO aortas (Fig. 5j, k). Accordingly, PWV demonstrated that the stiffness of NKO aorta was significantly reduced compared to WT mice that developed AAA (Fig. 5l). Altogether, these results indicated that VSMC niched in AAA milieu are subjected to abnormal cytoskeletal stress, unlike VSMC that comprise a healthy vascular wall environment. Therefore, our data imply that enhanced CSK tension of VSMC could have a causative role in the pathology of AAA.

### Mechanosensitive ion channel Piezo1 is increased in VSMC in AAA

**Fig. 6:**
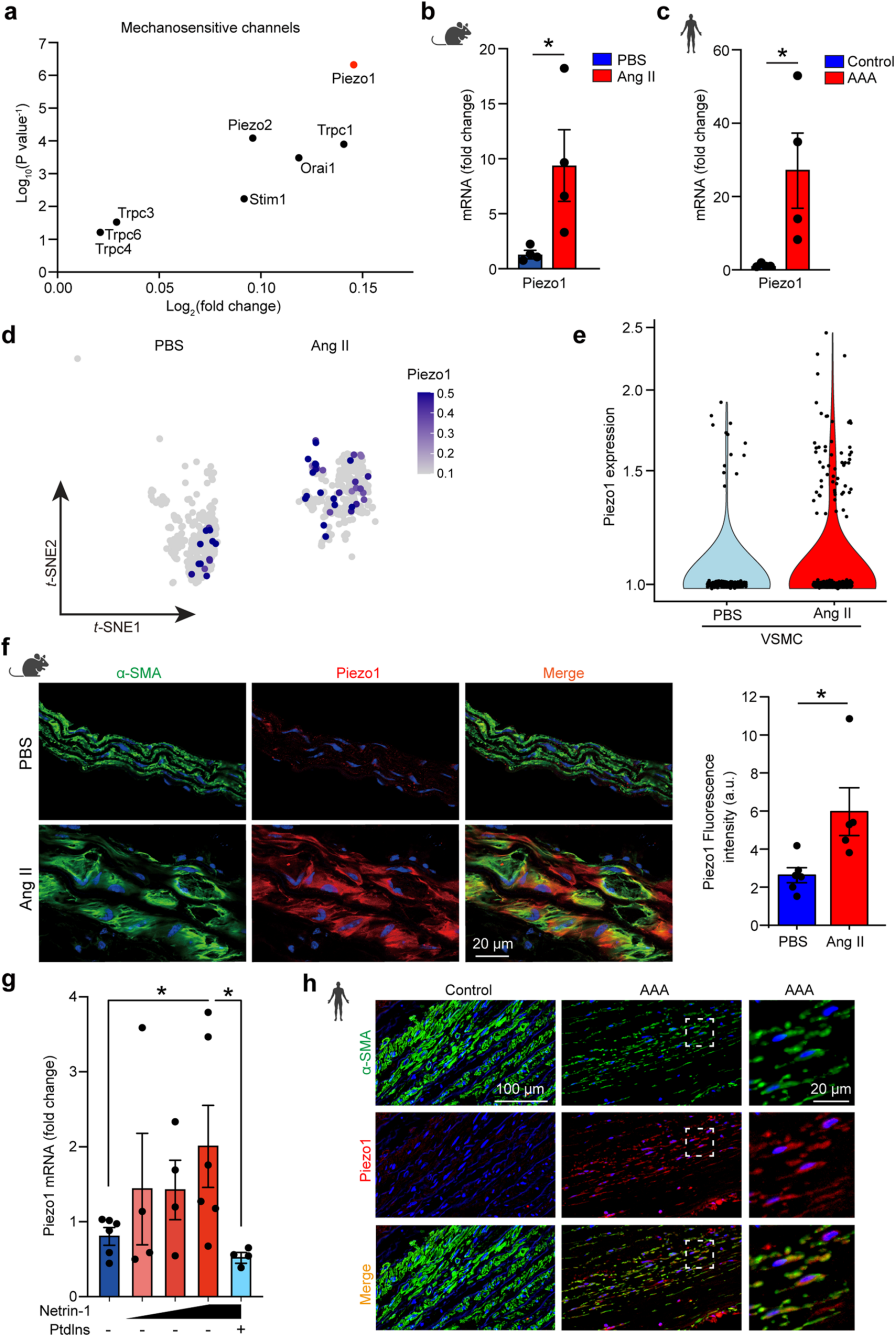
Mechanosensitive ion channel Piezo1 is increased in VSMC in AAA. **a** Dotplot representation showing differential expression (log_2_) and *P* value of mechanosensitive channel transcripts in AAA. Quantitative-RT-PCR (qPCR) analysis of *Piezo1* mRNA levels in mice (**b**) or human aortic specimens (**c**). *n* = 4 mice for **b** and *n* = 4 human aortic specimens for **c**. Two-sided Mann–Whitney test. *P = 0.0286 for both **b** and **c**. Data are represented as mean values ± SEM. **d** *Piezo1* mRNA expression in VSMC cluster in scRNAseq dataset and violin plot representation of Piezo1 expression (**e**); *n* = 4 per group. **f** Representative IF microscopy image of Piezo1 (red) and α-SMA (green) staining in aorta of mice and quantification. a.u., arbitrary unit. *n* = 6 mice aortic specimens for PBS and 5 for Ang II. Two-sided Mann–Whitney test. **P = 0.0087. Data are represented as mean values ± SEM. **g** qPCR analysis of *Piezo1* mRNA in VSMC stimulated with Netrin-1 (0.3, 0.625, or 1.25 µg/ml) or vehicle with or without PtdIns-(3,4,5)-P3 (25 µM) as indicated. *n* = 6, 4, 4, 6, 4 independent experiments, respectively. One-way ANOVA followed by uncorrected Fisher’s LSD test. *P = 0.0393 and 0.024, respectively. Data are represented as mean values ± SEM. **h** Representative IF microscopy images of Piezo1 (red) and α-SMA (green) in control and AAA aorta of human origin. Enlarged images of the boxed area with dashed lines in AAA aortic sections are shown in the right panel. *n* = 3 human aortic specimans per group. Nuclei are stained with DAPI (blue) in all IF images. Tissue sections in **f** and **h** were transversal sections. Mouse and human icons in **b**, **c**, **f**, and **h** are created with BioRender.com. Source data are provided as a Source Data file.

Since our experimental and theoretical models confirmed a rigidified state of VSMC in AAA, we further investigated the downstream molecular signals triggered by elevated α-actinin2. We first screened for mechanosensory signatures in VSMC by single-cell RNAseq of control and AAA aorta. Since we have previously demonstrated that Netrin-1 regulates Ca^2+^ flux in VSMC^23^, we focused on profiling mechanosensitive Ca^2+^ ion channels in our dataset. Interestingly, genes encoding for mechanosensitive ion channels with high affinity for Ca^2+^, Piezo1 was highly expressed in AAA tissues (Fig. 6a). Quantitative RT-PCR analysis confirmed *Piezo1* mRNA was increased by 10-fold in AAA aortas compared to non-diseased control tissues (Fig. 6b). A similar pattern of expression was observed in human specimens of AAA and control aortic tissue revealing increased expression of Piezo1 (Fig. 6c). Profiling of Piezo1 in cellular clusters revealed that its expression was concentrated in VSMC in experimental AAA (Fig. 6d, e). Immunofluorescence staining confirmed that Piezo1 protein levels were increased in AAA and co-localized with α-SMA positive VSMC in AngII induced AAA (Fig. 6f). Furthermore, Piezo1 mRNA expression was increased dose-dependently by recombinant Netrin-1 in VSMC and repressed in presence of α-actinin2 inhibitor, PtdIns(3,4,5)P3 (Fig. 6g) indicating a coordinated action of mechanical tension and non-mechanical stimulus (Netrin-1) in regulating Piezo1 expression in VSMC. Notably, immunofluorescence staining revealed marked expression of Piezo1 in VSMC in human aneurysmal sections compared to a minimal expression pattern detected in non-aneurysmal control tissues (Fig. 6h). Altogether, these findings indicate that Piezo1 is a novel downstream mechano-effector of Netrin-1 in AAA.

### Netrin-1 regulates intracellular Ca^2+^ influx via Piezo1 in VSMC

**Fig. 7:**
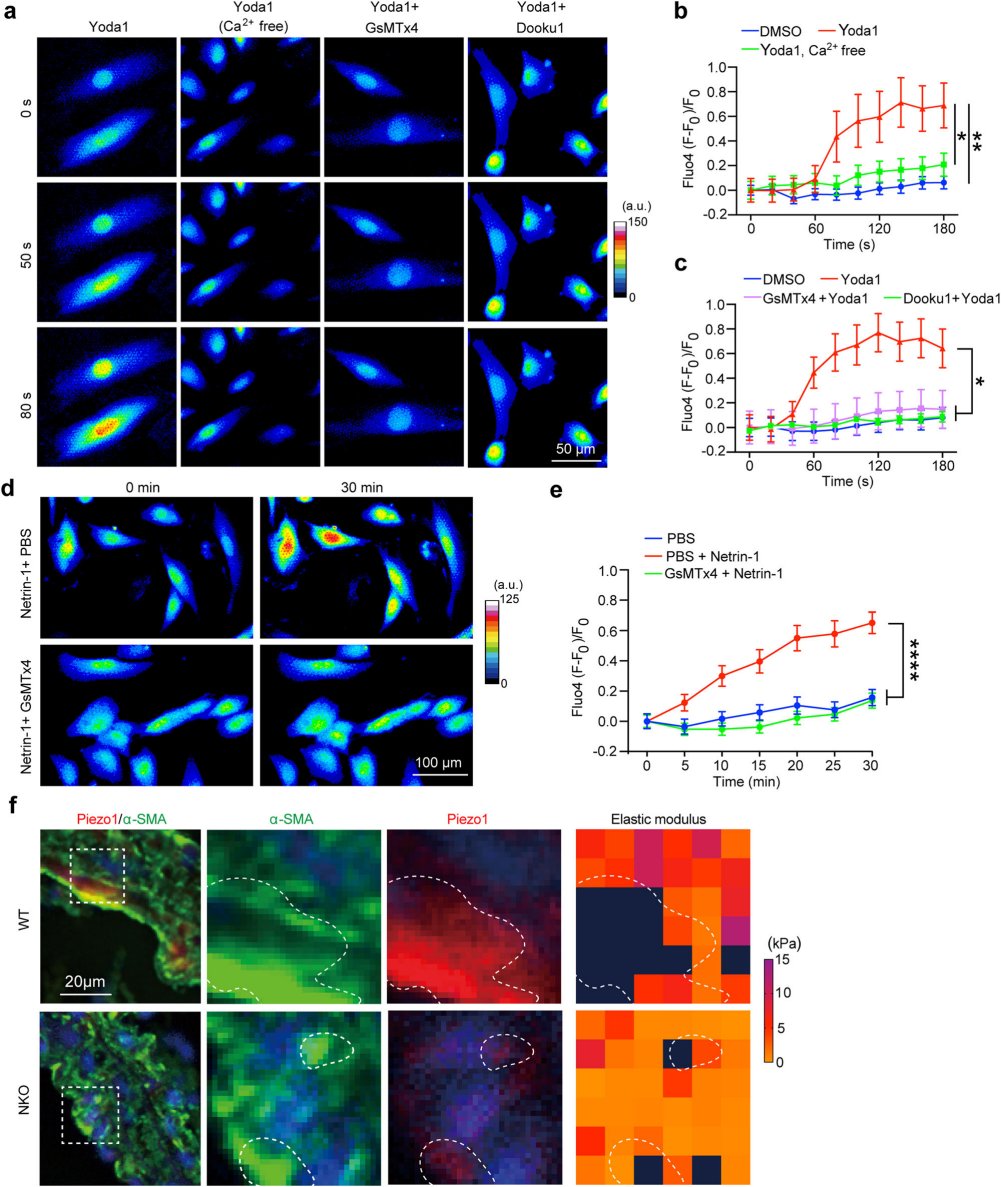
Netrin-1 regulates intracellular Ca^2+^ influx via Piezo1 in VSMC. **a** Representative images of intracellular Ca^2+^ signal in VSMC treated with reagents and time period as indicated. The signal intensity scale is indicated on the right. a.u., arbitrary unit. **b**, **c** Time course quantification of Ca^2+^ flux in VSMC treated as indicated. For **b**, *n* = 5, 8, and 5 independent measurements for DMSO, Yoda1 Ca^2+^ free, and Yoda1, respectively. One-way ANOVA followed by uncorrected Fisher’s LSD test. *P = 0.025 and **P = 0.0082. For **c**, *n* = 5, 5, 8, and 11 independent measurements for DMSO, Yoda1, GsMTx4 +Yoda1, and Dooku1 + Yoda1, respectively. One-way ANOVA followed by Turkey’s multiple comparisons test. *P = 0.0191 (DMSO vs Yoda1), *P = 0.0230 (Yoda1 vs GsMTx4 +Yoda1), and *P = 0.0321 (Yoda1 vs Dooku1 +Yoda1). Data are represented as mean values ± SEM and multiple comparisions tests are performed at *t* = 180 s for both **b** and **c**. **d**, **e** Representative images (**d**) and quantification (**e**) of Ca^2+^ levels in VSMC were treated as indicated. The signal intensity scale is indicated on the right of **d**. a.u., arbitrary unit. For **e**, *n* = 33, 24, and 26 cells for PBS, PBS + Netrin-1, and GsMTx4 + Netrin-1, respectively. One-way ANOVA followed by Turkey’s multiple comparisons test. ****P < 0.0001. Data are represented as mean values ± SEM. **f** IF staining of α-SMA (green) and Piezo1 (red) in aortic sections of WT or NKO mice. The dashed box indicates the magnified area of AFM and immunostaining. Piezo1 and α-SMA expression and elastic modulus in areas of interest are outlined. Nuclei are stained with DAPI (blue). Tissue sections in **f** were transversal sections. Source data are provided as a Source Data file.

We further evaluated the functional significance of elevated Piezo1 induced by Netrin-1 in VSMC. Microscopy images showed that stimulation of VSMC with Yoda1, a specific agonist of Piezo1^35,36^, significantly increased intracellular Ca^2+^ influx (Fig. 7a) which was maintained for 200 s in VSMC (Fig. 7b). This was mostly dependent on extracellular pools of Ca^2+^ since Yoda1-induced intracellular flux of Ca^2+^ was abrogated in conditions when the media was depleted of Ca^2+^ (Fig. 7a, b). In the presence of GsMTx4 peptide, an antagonist previously shown to block Piezo1 channels^37^, or a Yoda1 antagonist – Dooku1^38^, the increase of intracellular Ca^2+^ flux induced by Yoda1 was significantly reduced (Fig. 7a, c), suggesting that GsMTx4 can block mechano-activation of Ca^2+^ via Piezo1 in VSMC. Netrin-1 triggered a robust Ca^2+^ influx in VSMC but was mitigated in the presence of GsMTx4 (Fig. 7d, e). AFM microscopy revealed that areas enriched with Piezo1 in aortic sections of AAA mapped with territories displaying increased elastic modulus within the tissue. Piezo1 expression and elastic modulus within NKO aortic sections were weaker (Fig. 7f). This was consistent with α-actinin2 and elastic modulus patterns observed between WT and NKO sections. Altogether, these findings suggested a central role for Netrin-1 in fueling Ca^2+^ supply to VSMC by mechanically powering Piezo1 channels.

### Antagonizing Piezo1 prevents AAA development

**Fig. 8:**
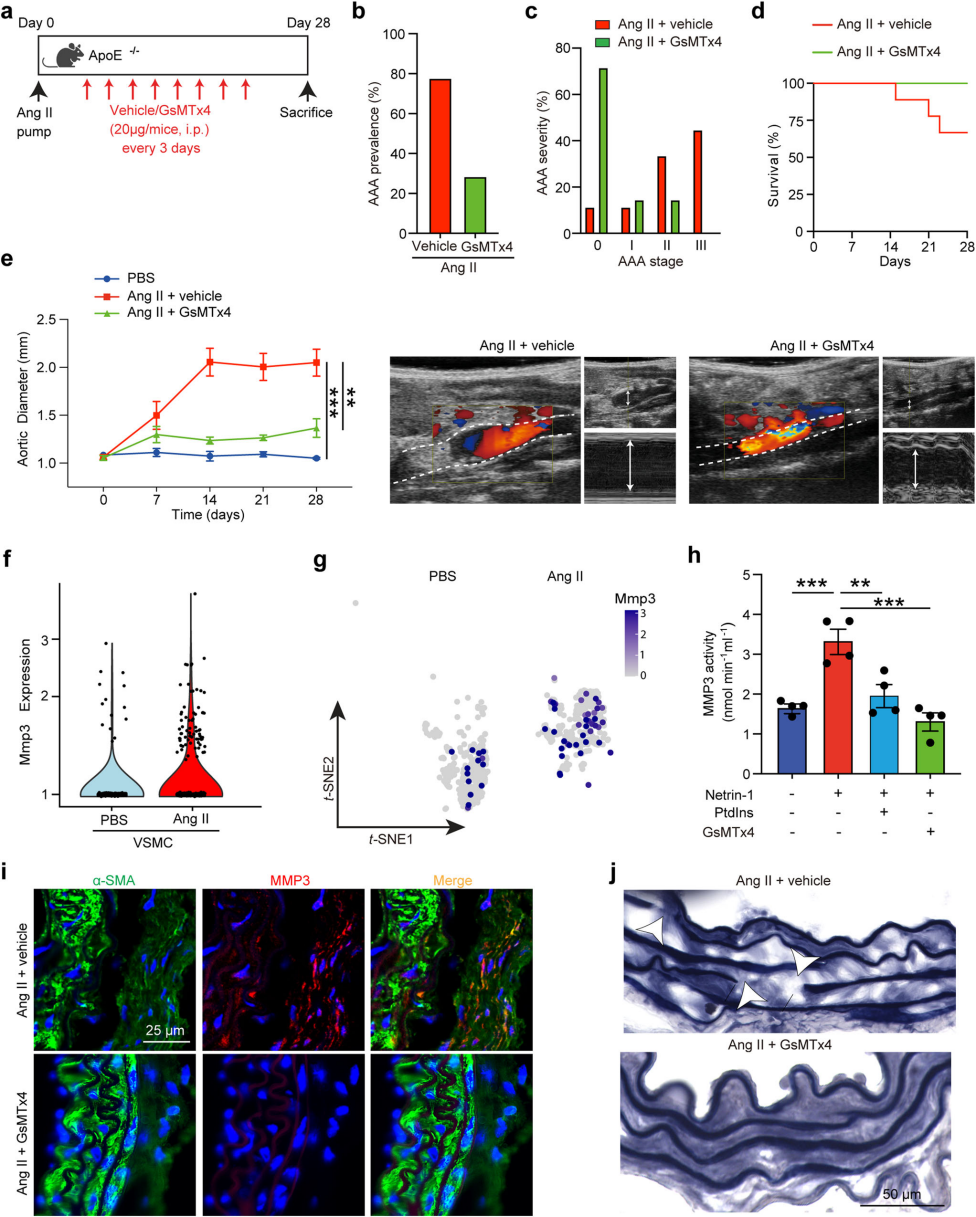
Antagonizing Piezo1 activation prevents AAA development. **a** Schematic representation of GsMTx4 treatment during AAA induction. i.p., intraperitoneal. AAA prevalence (**b**), AAA severity score (**c**), and survival rates (**d**) in mice treated as indicated. **e** Measurements of maximum aortic diameter over time (left) and representative color Doppler ultrasound images (right) of aortic flow as indicated. The dotted line delimitates the aortic wall. Arrows show maximum diameter. *n* = 3, 6, and 7 mice for PBS, Ang II + vehicle, and Ang II + GsMTx4, respectively. One-way ANOVA followed by Turkey’s multiple comparisons test. **P = 0.0017 and ***P = 0.0005. Data are represented as mean values ± SEM. **f** Violin plot of Mmp3 mRNA expression in VSMC and **g** its t-SNE distribution in single-cell RNA seq dataset. **h** Mmp3 activity assay in VSMC stimulated with Netrin-1 with or without GsMTx4. *n* = 4 per group. **i** Immunofluorescence staining of Mmp3 (green) and α-SMA (red) in aortas of mice exposed to Ang II, vehicle or GsMTx4. *n* ≥ 3 per group. One-way ANOVA followed by Turkey’s multiple comparisons test. **P = 0.0037 and ***P = 0.0007 (Control vs Netrin-1) and 0.0001 (Netrin-1 vs Netrin-1 + GsMTx4). Data are represented as mean values ± SEM. Nuclei are stained with DAPI (blue). **j** Verhoeff-Van Gieson elastin staining in aortic sections of indicated mice. Arrows indicate elastin breaks. *n* = 3 independent experiments per group. Tissue sections in **i** and **j** were transversal sections. Mouse icon in **a** is created with BioRender.com. Source data are provided as a Source Data file.

On the basis of the experimental and theoretic results reported above, we postulated that antagonizing Piezo1 would impede ECM destruction and curb AAA development. We therefore exposed mice to continuous subcutaneous infusion of Ang II (Fig. 8a) or peri-adventitial elastase infusion (Supplementary Fig. 6a) and subjected them to vehicle or GsMTx4 treatment. The incidence of AAA was decreased with GsMTx4 treatment, consistent with reduced disease severity (Fig. 8b, c and Supplementary Fig. 6b, c) and increased survival rate (Fig. 8d). Doppler ultrasound images captured enlarged aorta in vehicle-treated mice, but not in those exposed to GsMTx4 (Fig. 8e and Supplementary Fig. 6d). Quantification of maximum aortic diameter confirmed that Ang II treatment increased aortic dilatation, which was reduced in the presence of GsMTx4 (Fig. 8e and Supplementary Fig. 6e). These results suggested that negatively interfering with Piezo1 activation rescued mice from developing AAA. We previously demonstrated that Netrin-1 commands the release of proteolytic enzyme, MMP3, by VSMC to drive elastin degradation in AAA^23^. Notably, single-cell RNAseq analysis demonstrated that MMP3 was likewise enriched in VSMC populations in AAA (Fig. 8f, g). Stimulation of VSMC with recombinant Netrin-1 induced MMP3 activity and was blunted in the presence of GsMTx4 or PtdIns, thus validating that Netrin-1 regulates MMP3 via Piezo1 and α-actinin2 in VSMC (Fig. 8h). Immunofluorescence staining confirmed the reduced expression of MMP3 in VSMC in aortic section of mice treated with GsMTx4 compared to controls (Fig. 8i). This was consistent with reduced elastin fiber damage characterized by thicker and unfragmented fibers, in vascular wall of mice treated with GsMTx4 in accordance with non-diseased phenotype (Fig. 8j). Similar reduced MMP3 and elastin fiber fragmentation were observed in elastase infused AAA mice treated with GsMTx4 (Supplementary Fig. 6f, g). Activation of Piezo1 with Yoda1-induced MMP3 activity which was repressed in presence of GsMTx4 in VSMC (Supplementary Fig. 6h). These results were validated by single-cell RNA sequencing of elastase-induced AAA demonstrating the coupling between α-actinin2, Piezo1, and MMP3 in AAA (Supplementary Fig. 6i). Furthermore, we validated our key findings in human VSMC, demonstrating that recombinant Netrin-1 can modulate VSMC stiffness and that Piezo1 activation is required to stimulate MMP3 activation (Supplementary Fig. 7). Together, these results invoke that suppressing Piezo1-mediated mechanotransduction hinders proteolytic damage of the aorta and is likely to decelerate AAA growth in patients should such drug-based approach be brought to the clinical setting.

## Discussion

In this study, we mapped a novel mechano-molecular trajectory connecting AAA-prone signals that outset the activation of mechanosensitive ion channel, Piezo1, to fuel matrix degradation during AAA progression (Supplementary Fig. 8). We observed increased stiffness of the vascular framework in aneurysmal conditions where cellular and extracellular constituents are damaged. This biomechanical pattern was reciprocated in VSMC isolated from AAA aorta or in the presence of AAA-promoting signal Netrin-1. Notably, we demonstrate that pathological mechanical load of VSMC was fueled by increased CSK crosslinking fueled by α-actinin2 thereby mitigating intracellular mechanotransduction signals that powered the opening of Piezo1 channels. These results are consistent with other studies that have demonstrated elevated stiffness and dysfunctional mechanosensing in aneurysmal aortic tissue^39–41^. Our findings extend on this knowledge by invoking that VSMC are instrumental bearers of defective mechanosensing during AAA and identify Piezo1 as a novel causative factor for AAA.

It is unsurprising that epidemiologic studies have established associations between increased stiffness and AAA^39,42^ given that AAA affects almost exclusively the aged population (≥65 years) and that stiffness of the aorta culminates with aging^43,44^. Further comprehensive studies have dissected that increased mechanical stress not only positively correlated with risk of rupture^45^ but also coincided with the rate of expansion of AAA^46^. These clinical findings were recapitulated in experimental AAA modeled by elastase infusion which demonstrated that elevated segmental aortic stiffening could be a stimulus that initiates AAA growth^47^. Our findings of increased stiffness are consistent with the latter study. Here, we further show that, Piezo1-driven mechanosensing is a pathological burden in two experimental models of AAA. Alleviating this mechanical stress protected mice from developing pathological vascular remodeling and reduced AAA incidence. This suggested that elevated mechanical load is an overlapping feature between these two experimental models and is likely an intrinsic trait of AAA.

In contrast to the extensive study of unicellular “static” biophysical properties and their relation to cell status, exploring how cells dynamically respond to cellular architecture and pathological mechanical stimuli has been a challenge especially in cells that are embedded in pulsatile tissues such as the aorta. We engineered a sophisticated system to bypass these challenges by allowing the monitoring of time-variant molecular activities and CSK dynamics in response to transient perturbations of VSMC. Using our ultrasound tweezers-based integrative micromechanical system, we revealed both the progressive and instantaneous mechanosensation behaviors of VSMC to transient mechanical stimulus. We show that compromised mechanosensation of VSMC is acquired gradually as AAA develops. Similar mechanic allosteric shifts were observed in non-pathological VSMC treated with Netrin-1. This adds on to our previous studies demonstrating that mechanical perturbations are integrators that determine distinct mechanophenotypes in diseases^30,31^. Such changes in mechanosensation of VSMC can be attributable to the overexpression of α-actinin2 which causes a more solid-like property of CSK. Combined with a sensitive spectral analysis, we can capture the time-evolution of VSMC CSK and the decay in cellular viscoelastic properties during AAA development. Moreover, AFM revealed that VSMC within AAA tissue are stiff and PWV validated increased aortic tissue stiffness consistent with results obtained with our micromechanical system. Our results indicate that measuring VSMC mechanosensation behaviors might be used as a new biophysical marker to evaluate AAA progression. This is also demonstrated in our theoretical modeling. In the modeling, we mimic the development of AAA by simply changing the viscoelastic properties of VSMC due to the increasing expression of α-actinin2. To further elucidate the “spontaneous property” of VSMC mechanosensation, we modeled a mechanical actuator that is produced by the activation of myosin, but its work will largely depend on the deformability of CSK. The modeling not only reflects the “non-invasiveness” of our in vitro micromechanical system for perturbating single cells, but also accurately probed the mechanism of VSMC defective mechnosensation in AAA. Combining experimental measurments and theoretical modeling, our analysis of VSMC mechanosensation can provide insights into the pathological molecular machinery that drives the compromised force generation ability of VSMC in AAA and may help to provide guidelines in selecting pharmaceutical candidates for inhibiting AAA.

Our results demonstrate that in the advanced stages of AAA, the accumulation of secreted molecules such as Netrin-1 provoked the increase of VSMC cell membrane tension by finetuning actin fiber crosslinking through α-actinin2, thereby powering Piezo1 activation in VSMC. This is consistent with other studies that demonstrated that Netrin-1 regulated macrophage motility^48^ and neuron outgrowth^49^ by modulating actin filament machinery through FAK activation. We therefore demonstrated a novel concept illustrating that non-mechanical stimulus (secreted Netrin-1) released by macrophages can coordinate with increased tension wall generated by anatomically deformed aorta to activate the expression and responsiveness of Piezo1 in VSMC. We also demonstrated that Piezo1 is transcriptionally regulated in tensed VSMC and AAA tissue. This most likely reflects a feed-forward mechanism initiated by increased vascular tension and Netrin-1 that sustains transcriptional regulation of the gene encoding for Piezo1^50^. Importantly, this suggested that in homeostatic conditions, during which VSMC are niched within complex mechanically active microenvironments and subjected to concentric pressure load, the expression of Piezo1 is repressed basally to avoid overactivation of Ca^2+^ signals. Indeed, we observed that Piezo1 was weakly expressed at baseline in VSMC within non-diseased aortic tissue. This feedback regulatory mechanism seems to be compromised with the surge of Netrin-1 stemming from arterial macrophages in the microenvironment of VSMC. Therefore, breaking this pathological circuitry by targeting Piezo1 is a highly desirable goal as it would allow for the elimination of signals that promote a continuum in matrix degradation permissive to AAA development. We show that treatment of mice prone to develop AAA, using two experimental models of AAA, with GsMTx4 prevented AAA incidence by refraining the influx of Ca^2+^ in VSMC, a necessary co-activator of ECM-degrading enzyme MMP3. Our in vitro studies show that Netrin-1-induced Ca^2+^ influx and MMP3 activity can be repressed in the presence of GsMTx4. Similar Ca^2+^ influx patterns were induced in VSMC by specific activators of Piezo1, Yoda1. As such, attempts to block Ca^2+^ channels to prevent AAA has only been successful when combined with additional therapy such as statin inhibitors^51^. Notably, evidence suggests that treatment of patients undergoing endovascular repair to prevent AAA rupture, with a Ca^2+^ channel blocker, demonstrated beneficial outcomes on the repair intervention as evaluated by AAA sac shrinkage^52^. These studies indicated that attempts to repress Ca^2+^ as monotherapy or in combination with surgical interventions are desirable approaches to curb AAA development. Indeed, our results show that targeting upstream importer of Ca^2+^ that are selectively upregulated during disease development in VSMC could provide a specific target in refraining exaggerated matrix-degrading enzyme activation in AAA. Importantly, AAA is often asymptomatic and, in most cases, it is detected by accident following imaging of the chest. Patients already have established AAA when detected, suggesting likely activation of Piezo1. We also identified elevated Piezo1 expression in VSMC in human AAA tissue and demonstrate that human VSMC reciprocate the mechanosensitive behaviors observed in murine cells in vitro. We speculate that artificially mapping mechanical behaviors of cells within AAA combined with monitoring Piezo1 activity could be promising strategies to predict the development of AAA.

Taken together, our study provides sophisticated models that revealed novel insights into the mechanism of Piezo1-mediated pathological mechanosensation in AAA. However, additional studies should be performed to provide evidence that supports the potential strategy to use Piezo1 antagonists to decelerate or stabilize AAA growth. The side effects of such strategies should be carefully cataloged given the potential systemic responses that might be elicited with targeting Piezo1. Accordingly, developing nanomedicine strategies to locally deliver Piezo1 antagonists at the site of AAA in combination with surgical interventions could provide attractive opportunities to circumvent these side effects.

## Methods

### Mice

Mice used in our experiments, including C57BL/6J (WT), LysMcre, and ApoE^−/−^ mice were bought from Jackson Laboratories. Ntn1^flox/flox^LysMcre^+/−^ and control Ntn1^flox/flox^LysMcre^−/−^ mice were generated as described previously^23^. Mice were bred in a pathogen-free facility and provided a standard chow diet unless otherwise stated. In each experimental group, mice were randomly distributed, and no predetermination of sample size was conducted by statistical method. The US Department of Agriculture Animal Welfare Act, the Public Health Service Policy for the Humane Care and Use of Laboratory Animals and the New York University School of Medicine’s Institutional Animal Care and Use Committee approved all experimental procedures which were conducted according to their guidelines.

### AAA induction in mice

Procedures described previously^23,53^ by us and others were used to induce AAA in mice. Briefly, 8-week-old male ApoE^−/−^ mice were implanted with Alzet osmotic pumps (#2004; Durect Corporation) sub-cutaneously for continuous 28-day delivery of PBS (control) or angiotensin II (H-1705, Bachem) at 1 μg/kg/min. For Ntn1^flox/flox^LysMcre^+/−^ and Ntn1^flox/flox^ LysMcre^−/−^ littermates on C57BL/6J background, AAA was induced by increasing their cholesterol through exposure of adeno-associated virus vector (AAV) overexpressing proprotein convertase subtilisin/kexin type 9 (PCSK9) by intraperitoneal injections contiguous with a Western diet (WD; 21% [wt/wt] fat, 0.3% cholesterol; Research Diets) feeding for 2 weeks prior to osmotic pump implantation. Mice developed AAA when maintained on Western diet throughout angiotensin II perfusion as described previously^54^. For piezo1 inhibition experiments, ApoE^−/−^ mice received intraperitoneal injections of GsMTx4 (STG-100, Alomone Labs), 20 µg/mice every 3 days. Blood pressure was measrued by tail cuff using the CODA machine (Kent Scientific Corporation) as per the manufacturer’s instructions. Briefly, mice were restrained and placed on a warmed surface. A tail-cuff was placed at the foremost top part of the tail and blood pressure was measured at baseline and after Ang II pump implantation. Multiple recordings were made and mean of systolic blood pressure is shown (Supplementary Fig. 9). Severity of AAA was evaluated at sacrifice based on gross morphological aortic alterations as per published guidelines^55^: Aortas without dilation and sign of disease were scored as stage 0. Dilated aortas and aortas presenting a bulging without thrombus were scored as stages I and II, respectively. Bulbous aortas with visible thrombus were scored as stage III. In the case of mice death prior to experimental procedure completion, a necropsy was performed to assess the cause of death; rupture was characterized by the presence of an intraperitoneal hemorrhage and graded stage III.

### Doppler ultrasound imaging

Aortic diameter and pulse wave velocity (PWV) was assessed weekly using a Vevo 2100 ultrasound imaging platform (FUJIFILM VisualSonics)^24^. Mice were anesthetized using 2% isoflurane inhalation, placed on a supine position and the abdominal region was shaved and coated with aquasonic ultrasound transmission gel (NC9861677, Parker Laboratories) before positioning the acquisition probe. Mice heart rate and basal temperature were monitored throughout the ultrasound imaging procedure. Color mode was activated to detect the renal region. M mode was activated and aortic diameter was acquired on the suprarenal region for the Ang2 model, and on the infrarenal region for the elastase model. PW mode measurements were applied to the same regions and the pulse wave was measured with an angle of 20°. Velocity Time Integral was automatically determined by the machine in Analyze mode. The PWV velocity was then calculated by multiplying the velocity time integral (VTI) by the aortic circumference (PWV = VTI × *π* × (aortic diameter/2)^2^. Measurements were analyzed blinded to the experimenter.

### Human samples

All protocols followed involving human subjects are compliant to the Declaration of Helsinki principles. The studies were conducted in accordance with regulations set forth and approved by the New York University Institutional Review Board (IRB). The study was authorized with IRB approval number i16-01807. Aortic aneurysmal tissues were collected from patients undergoing open aortic aneurysm repair. Informed consent was obtained from each subject prior to surgery. Healthy control tissues were obtained from brain-dead multi-organ donors, and were provided by LiveOnNY organization (NewYork, NY). Informed consent for the use of the samples was obtained from the donors or their families. All tissues were macroscopically inspected, oriented and fixed prior to paraffin-embedding and sectioning.

### Cell culture

Mouse primary VSMC were isolated angiotensin II- or PBS-perfused C57BL/6J mice aortas that were freshly dissected, as previously established^56^. Peri-aventitial fat was carefully removed and the dissected aortas were *processed* for *digestion* for 10 min at 37 °C in an enzymatic mix composed of type II collagenase (10 mg/ml, C6885, Sigma Aldrich) and elastase (1 mg/ml, LS002292, Worthington Biochemistry). After removal of the adventitia under a dissecting microscope, the samples were further digested for 50 min in the enzymatic mix. Single-cell suspensions were purified through a 70 µm cell-strainer. Cells were grown in DMEM supplemented by 10% fetal bovine serum and 1% penicillin-streptomycin until confluence and used for assays as outlined in the manuscript. VSMC purity was verified by content of alpha-actin by flow cytometry and confirmed with additional positive for VSMC marker (smooth muscle myosin heavy chain (SM-MHC) and negative for fibroblasts marker (Fibroblast surface marker (FSP)) and endothelial cell marker (CD13) by immunofluorescence staining (Supplementary Fig. 10). Human VSMCs were purchased from Lonza and maintained per manufacture instructions with SmGM-2 Smooth Muscle Growth Medium BulletKit (Lonza). For all experiments, primary VSMC were cultured for no more than three passages.

### Western blotting

The protein content of samples was collected in RIPA buffer (98065; Cell Signaling Technologies) and loaded onto a 10% Mini-PROTEAN TGX gel (#456-8034, Bio-Rad) for SDS-PAGE and transferred to PVDF membranes (#BR20160719, Bio-Rad) using a Trans-Blot Turbo Transfer System (Bio-Rad). Non-specific signals were blocked and membranes were probed using anti-α-actinin2 (1:500 dilution; 14221-1-AP, Proteintech) and anti-α-actin (sc-130656, Santa Cruz Biotechnology) antibodies. HRP conjugated secondary antibodies, goat anti-rabbit (1:5000 dilution; 7074P2, Cell Signaling), or goat anti-mouse (1:5000 dilution; A9917, Sigma Aldrich) were incubated with the membranes. Protein bands were revealed by applying Clarity Western ECL (#170-5060, Bio-Rad) and imaged on a ChemiDoc Imaging System (Bio-Rad). Mean band intensities normalized to control were quantified with ImageJ software.

### Membrane rigidity assay

Membrane rigidity was assessed using the Membrane fluidity kit (ab189819, Abcam) as per manufacturer’s instruction. Mouse VSMC were seeded in flat-bottom clear 96-well plates (5 × 10^4^ cells/well). Cells were stimulated for 24 h with recombinant Netrin-1 (1109-N1, R&D Systems) in the presence or absence of VIVIT peptide (MAGPHPVIVITGPHEE peptide, #3905, Tocris Biosciences, 1 µM), BAPTA (B1205, Invitrogen, 10 µM) or PtdIns­(3,4,5)­P3 (10007764, Cayman Chemical, 25 µM). All pharmacological inhibitors were added 1 h before Netrin-1 stimulation. Following treatment, cells were washed and stained for 20 min at 37 °C, 5% CO_2_, using the 10 µM Fluorescent Lipid Reading containing 0.08% Pluronic F127 in perfusion buffer, all provided in the kit. Cells were then washed twice and fluorescence was measured immediately using a FlexStation 3® Multi-Mode Microplate Reader (Molecular Devices). Fluorescence was measured at 400 and 460 nm and data was expressed as the ratio of values captured at 460 nm and 400 nm wavelengths.

### RNA sequencing

RNA sequencing dataset of WT and NKO previously published by our group^23^ was re-analyzed by using the DESEQ2 package from Bioconductor running on R statistical program. For single-cell RNA sequencing, control and AAA aortas were digested in a single suspension as described above. Following the digestion, viable cells were enriched using a dead cell removal magnetic kit (130-090-101, Miltenyi Biotech) and cellular viability, assessed using 0.4% Trypan blue (1450013, Bio-Rad) on an automated cell counter (TC20, Bio-Rad). Hastagged samples were incubated with oligo-tagged antibodies (TotalSeq-B, Biolegend) according to manufacturer’s instructions. 15,000 of viable cells per sample were loaded on a 10x Genomics Chromium instrument to obtain individual gel beads in emulsion (GEMs). Chromium Single Cell 3′ Library & Gel Bead Kit v2, PN-120237, the Single Cell 3′ Chip Kit v2 PN-120236 and the i7 Multiplex Kit PN-120262, (10x Genomics) were used for library preparation. Illumina HiSeq 4000 as 2 × 150 paired-end reads was used for sequencing (>90% sequencing saturation). Cell Ranger Single Cell Software Suite, version 1.3, was used to perform de-multiplexing, barcode and UMI processing, and single-cell 3′ gene counting (https://support.10xgenomics.com/single-cell-gene). Data analysis was performed using the ‘Seurat’ version 3.1.2 package, using R Studio Desktop, version 1.2.5033, and R, version R 3.0.1+. Quality control, metrics, data normalization, scaling, batch correction and dimension reductions were all performed using the Seurat package. Cells with mitochondrial transcript proportion <5% were kept for analysis, neighboring and clustering was performed on the most significant principal component analysis. The VSMC cluster, identified based on the enrichment in *Acta2* and *Myh11* specific markers, was used for differential expression in the control and AAA group. P values were adjusted using the Benjamini-Hochberg method for false discovery correction. Genes with an adjusted *P* value < 0.05 were considered as differentially expressed and used for enrichment analyses using Ingenuity Pathway Analysis (IPA). All scripts are available from authors upon reasonable request.

### Real-time quantitative PCR

Aortic samples were lyzed in TRIzol (15596026, Ambion, Life Technologies) and RNA isolation was performed using the Directzol RNA MiniPrep Kit (R2052, Zymo Research). Following reverse transcription using the cDNA Synthesis Kit (1708890, Bio-Rad), quantitative real-time PCR was performed with KAPA SYBR FAST qPCR Kits (KK4602, KAPA Biosystems) using a QuantStudio 3 Real-Time PCR System (Applied Biosystems). The primer sequence set used were: muPiezo1: Forward: TCATCATCCTTAACCACATGGTG, Reverse: TGAAGACGATAGCTGTCATCCA; huPiezo1: Forward: CAATGAGGAGGCCGACTACC, Reverse: GCACTCCTGCAGCTCGATGA; 28S: Forward: TGGGAATGCAGCCCAAG, Reverse: CCTTACGGTACTTGTTGACTATCG. Fold change differences were calculated after normalization to housekeeping gene by the comparative cycle method (2^−ΔΔCt^).

### Immunofluorescence staining and microscopy

Sectioning of frozen murine aortic samples conserved in Optimal Cutting Temperature Compound (OCT; 4585, Fisher Scientific) generated 7-µm-thick sections which were fixed in 10% formalin buffer prior to immunostaining. Sections prepared in paraffin were deparaffinized, processed for tissue rehydration and antigen retrieval as previously described^23^. VSMC were fixed in 4% paraformaldehyde (Electron Microscopy Sciences) for 30 min and then permeabilized with 0.3% Triton X-100 (Roche Applied Science) for 10 min at room temperature. Staining was performed by applying overnight incubation of primary antibodies: anti-α-actinin2 (#14221-1-AP, Proteintech), anti-Piezo1, (#15939-1-AP, Proteintech), or anti-alpha-smooth muscle actin (#48938S, Cell Signaling) (1:200 dilution each). Alexa Fluor 488 and 647-conjugated anti-IgG antibodies (Invitrogen) were used for fluorescent signal detection. Alexa Fluor 555 conjugated to phalloidin (Invitrogen) and 4’,6-diamidino-2-phenylindole (DAPI; Invitrogen) were used for visualization of actin microfilaments and nucleus respectively. Validation of the primary anti-Piezo1 antibody was performed to confirm the specificity (Supplementary Fig. 11). Images were acquired using a Zeiss LSM 710 confocal microscope (Carl Zeiss) through the Zeiss Efficient Navigation (ZEN) software (Carl Zeiss). Identical acquisition parameters were set to capture control and test samples. Learning Unsupervised Means of Spectra (LUMoS)^57^ method (ImageJ) was used for analysis of staining intensity of some markers of VSMC.

### siRNA transfection

Piezo1 knockdown was performed by siRNA tranfection to validate the specificity of the Piezo1 antibody ((#15939-1-AP, Proteintech) used in this study. Murine VSMC were seeded on coverslips in a six-well culture plate at the density of 5000 cells/cm^2^. After the 24-h culture, VSMC were transfected with either 2 μg/mL ON-TARGETplus Piezo-1 siRNA Reagents (L-061455-00-0005, Horizon Discovery, Waterbeach, UK) or 2 μg/mL ON-TARGETplus non-targeting control pool (D-001810-10-05, Horizon Discovery) using Lipofectamine RNAiMAX transfection reagent (13778030, Life Technologies) as per the manufacturer’s instructions. Piezo1 knockdown was validated by immunoflorescence and quantitative real-time PCR.

### Atomic force microscopy

Aortic samples were cryosectioned using Leica CM3050S cryostat and immobilized to glass-bottom dish (WPI’s FluoroDish™) precoated with poly-d-Lysine for 24 h, 8-μm-thick sections were used as previously described^58^. An MFP-3D-BIO Atomic Force Microscope (Asylum Research, Oxford Instruments, Santa Barbara, CA) was used to obtain stiffness from aortic tissues. A 6.1-μm spherical polystyrene bead AFM probe (CP CONT-PS-C, NanoAndMore Lady’s Island, SC) was used for all indentation measurements. The actual spring constant was 0.27 N/m. The calibration of Z-distance deflection and the cantilever stiffness was determined by InvOLS and thermal tune calibration was determined following the previous protocols^59^. The force-distance plot was recorded during 8 μm travel distance of the cantilever from the tissue medium to an indentation depth of ~500 nm into the aortic sample. We used a deflection set point of 5 nN and an indentation rate of 16 μm s^−1^ to probe the elastic modulus and minimize viscoelastic effects. Two to four two dimensional ≥6 × 6 square grids region (≥18 μm × 18 μm) were probed per aortic sample. The force-indentation curves were fit to the Hertz model^60^ for spherical tips utilizing the Asylum Research Software to determine the Young’s Modulus, with an assumed Poisson’s ratio value of 0.45 for the aortic sample. The determined Young’s Modulus of each grid in the square region was then exported from the Asylum Research Software for further analysis. Average values from the measured Young’s Modulus data points were used to define the average Young’s Modulus of the region. An inverted microscope (Zeiss Axio Observer Z1) was used to acquire brightfield images of the aortic tissues to localize the region for the stiffness measurements. To correlate the measured Young’s Modulus with the level of α-actinin2 in the same region of the aortic tissue, immunofluorescence staining for α-actinin2 was performed for each aortic sample as described in the method “Immunofluorescence staining and microscopy”. To keep the AFM and microscopy segments matched, brightfield images were recorded when performing AFM and were used to align the sample for α-actinin2 imaging and mean fluorescence intensity quantification.

### Traction force measurement using micropillar array

VSMC CSK contractility was measured using the previously described PDMS micropillar array^61^. PDMS Micropillar array was fabricated through a double-molding process. A silicon master mold with a micropillar of ~7 µm height was first fabricated by standard photolithography and deep reactive ion etching. A negative PDMS mold was constructed by mixing 1:10 ratio PDMS prepolymer to the silicon mold and baked at 110 °C for 30 mins. The negative PDMS mold was peeled off the silicon mold and silanized with tridecafluoro-1,1,2,2,-tetrahydrooctyl)−1-trichlorosilane (Sigma-Aldrich) overnight in vacuum. To generate the PDMS micropillar array, a drop of 1:10 ratio PDMS was added to the silanized PDMS negative mold and covered with oxygen plasma (350 W, PlasmaEtch) treated cover glass. The “Cover glass-PDMS-negative mold” sandwich structure was cured in an oven heated at 110 °C. Cover glass with PDMS micropillar array was peeled off from negative mold and sonicated in 100% ethanol to restore collapsed micropillar. The PDMS micropillar array was dried and mounted on a petri dish with a 13 mm hole at the bottom. Prior to CSK contractility measurements, the PDMS micropillar array was primed with fibronectin (50 μg/ml; Sigma-Aldrich) and Alexa-Fluor 647-conjugated fibrinogen (25 μg/ml; Life Technologies) by microcontact printing so as to label the tip of pillar necessary for imaging as previously shown^62^. VSMC were cultured on the micropillar array overnight prior to mechanical stimulation and analysis. CSK contractility was analyzed by quantifying the deflection of micropillar using the Cellogram^63^ and custom-developed MATLAB programs (Mathworks).

### Scanning electron microscopy

VSMC were cultured on micropillar overnight. The samples were then washed three times with 50 mM Na-cacodylate buffer (pH 7.3; Sigma-Aldrich) and fixed for 1 h with 2% glutaraldehyde (Electron Microscopy Sciences) in 50 mM Na-cacodylate buffer. Fixed samples were dehydrated using a graded series of ethonal concntrations. Samples were first immersed in 30%, 50%, 70%, 80%, and 90% of ethanol for 10 mins, respectively, and then dehydrated in 100% of ethanol three times, each time for 20 mins. The dehydrated samples were dried using a super critical point dryer (Samdri®-PVT-3D, Tousimis) according to the instruction of manufacturer. To perform scanning electron microscopy (SEM), dried samples were mounted on stubs and sputtered with gold palladium for 15 s, observed and imaged under a Hitachi S-3400N Ultra-High Resolution SEM machine (Hitachi High Technologies America).

### Mechanical stimulation of VSMC

Mechanical stimulation using ultrasound tweezers for VSMC instantaneous mechanosensation was performed as previously described^29,30^. VSMC were functionalized with Targestar™-SA lipid microbubbles from Targeson (San Diego, CA) through RGD-integrin-CSK linkage. To functionalize the microbubble surface with RGD peptide 1 µL of microbubbles solution (3 × 10^9^/mL) was mixed with 4 µL of biotinylated Arg-Gly-Asp (RGD) peptide (2 mg/mL, Peptides International) for 20 min at room temperature. Cell culture medium was removed from the VSMC dish and replaced with 30 μL of the diluted microbubble-RGD added to the center of the micropillar array. The micropillar array was then flipped over in a cell culture incubator for 10 min to allow binding of microbubbles to VSMC. Unbound microbubbles were washed away with the cell culture medium. VSMC attached to microbubbles were used for ultrasound tweezers stimulation.

To generate ultrasound pulses for stretching microbubble, a 10-MHz ultrasound transducer (V312-SM, Olympus) was used and driven by a function generator (Agilent Technologies 33250 A) and a 75-W power amplifier (Amplifier Research 75A250). The ultrasound transducer was then calibrated and fixed at a 45° angle to the cell surface plane. The distance between the transducer and VSMC was calibrated to be 11.25 mm (Rayleigh distance) by using an Olympus pulser receiver. Ultrasound pulse was applied at a frequency of 1 Hz for 10 s on each VSMC with a microbubble attached. Deflection of the micropillar array underneath VSMC was continuously imaged to record the dynamics of CSK contractility and quantified as described above.

### Spectral analysis of VSMC force dynamics

To perform spectral analysis of VSMC force dynamics, the quantified VSMC real-time CSK contractility was sampled upon discretized windows which was generated by a previously developed algorithm^32^. We subsequently sampled time series of local force dynamics on each window to analyze localized force fluctuation. Based on the Empirical mode decomposition (EMD) of Hilbert-Huang transform^64,65^, we decompose the localized force dynamics into several analytical elementary signals, so called as intrinsic mode functions (IMFs). We then computed the instantaneous frequency spectra through Hilbert transform for all IMFs in all sampled windows, with central frequency hierarchically spanning from high to low values corresponding to IMF1-5. We also computed the frequency weighted quadratic amplitude of all IMFs to reflect the instantaneous energy consumed by the entire cell^32^. The temporal force response ratio is defined as $1-{e}^{t/{T}_{c}}$ where ${T}_{c}$ is the characteristic adaptation time that is equal to the reciprocal of IMF central frequency^33^.

### Biophysical modeling of VSMC instantaneouse mechanosensation

In the KVM model, the Kelvin–Voigt element, is modeled as a viscoelastic material consisting of elastic stress fiber (SF) with an elastic modulus of ${K}_{{{{{{{\mathrm{SF}}}}}}}}$ and a viscous component with the viscosity of $\eta$. The actin CSK of cells consists of filamentous actin that is crosslinked by actin crosslinking proteins^66^. As it has been experimentally shown that increasing crosslinker concentrations can directly increase CSK network stiffness and elasticity^67^ and loss of connectivity has also been shown to reduce the elasticity of the CSK network^68^, we therefore modeled the elastic modulus ${K}_{{{{{{{\mathrm{SF}}}}}}}}$ to be proportional to the amount of α-actinin2 and is described as ${k}_{{{{{{{\mathrm{sf}}}}}}}}{C}_{\alpha }$, where ${C}_{\alpha }$ represents the amount of α-actinin2 for a VSMC; ${k}_{{{{{{{\mathrm{sf}}}}}}}}$ is a scaling factor. To specifically study the change of VMSC CSK tension arising from contraction of myosin, the contractile myosin (CM) element is modeled in a series configuration with the viscoelastic CSK structure^34,69^. The KVM configuration can be further connected to an elastic substrate, which is the micropillar array (${K}_{{{{{{{\mathrm{pillar}}}}}}}}$) used to experimentally measure the change of CSK tension during the instantaneous mechanosensation process. As the KMV configuration is modeled in series, the Kelvin-Voigt element and the contractile myosin share the same tension, which is equal to the measured mechanical force by micropillar array,

1 $$F={K}_{{{{{{{\mathrm{pillar}}}}}}}}\Delta {L}_{{{{{{{\mathrm{pillar}}}}}}}}={K}_{{{{{{{\mathrm{SF}}}}}}}}\Delta {L}_{{{{{{{\mathrm{SF}}}}}}}}+\eta {\dot{L}}_{{{{{{{\mathrm{SF}}}}}}}}={F}_{{{{{{{\mathrm{CM}}}}}}},{{{{{{\mathrm{effective}}}}}}}}$$

where $F$ is the VSMC CSK tension measured by micropillar array, which is decided by the elastic modulus (${K}_{{{{{{{\mathrm{pillar}}}}}}}}$) and the deflection *(*$\Delta {L}_{{{{{{{\mathrm{pillar}}}}}}}}$) of the micropillar; ${L}_{{{{{{{\mathrm{SF}}}}}}}}$ is the length of the modeled stress fiber. $\Delta {L}_{{{{{{{\mathrm{SF}}}}}}}}$ is the deformation of stress fiber as a result of myosin sliding, where for ${\dot{L}}_{{{{{{{\mathrm{SF}}}}}}}}$, the dot indicates the time derivative as required for calculating stress of a viscoelastic material; ${F}_{{{{{{{\mathrm{CM}}}}}}},{{{{{{\mathrm{effective}}}}}}}}$ is the contractile force due to the effective sliding of activated myosin depending on the deformation of the CSK. To investigate the effect of change in CSK properties, herein the elevated expression of α-actinin2 in affecting cell force generation, we define *F*_CM,max_ as an instantaneous mechanical actuator that tends to deform CSK. To determine the magnitude of the mechanical actuator that deforms CSK, *F*_CM,max_ was modeled to be directly proportional to the maximum amount of activated myosin (*C*_M,max_), which is related to Ca^2+^ influx upon mechanical stimulus, *F*_CM,max_ ∝ *k*_m_*C*_m.max_, *k*_m_ is a scaling constant. As we did not see obvious difference of change of maximum Ca^2+^ influx upon mechanical stimulus for VSMC isolated from mice at different stages of AAA, *C*_M,max_ was kept constant and thus *F*_CM,max_ is constant. However, not all activated myosin contributed to the same amount of deformation of CSK with different viscoelastic properties, which results in different ${F}_{{{{{{{\mathrm{CM}}}}}}},{{{{{{\mathrm{effective}}}}}}}}$_._ To determine the deformation of CSK with different viscoelastic properties under the same mechanical actuator *F*_CM,max_, we modeled the deformation of CSK to closely follow a power law over time with an exponent *β* that reflects the viscoelasticity of CSK, $\Delta {L}_{{{{{{{\mathrm{SF}}}}}}}}\propto {t}^{\beta }$. The deformation is also related to the magnitude of CSK tension by activated myosin, hence $\Delta {L}_{{{{{{{\mathrm{SF}}}}}}}}={k}_{{{{{{\mathrm{m}}}}}}}{C}_{{{{{{\mathrm{m}}}}}},{\max }}{t}^{\beta }$. A material with as larger *β* indicates more viscous and smaller *β* indicates more elastic. We can then express *β* as ${k}_{\beta }\frac{\eta }{{K}_{{{{{{{\mathrm{SF}}}}}}}}}$, where *k*_*β*_ is a scaling factor. At the same time, more expression of α-actinin2 indicates a more elastic material and $\beta =\frac{{k}_{\beta ,a}}{{C}_{\alpha }}$, where ${k}_{\beta ,\alpha }$ is a scaling factor. Hence, we can model the tension accumulation in CSK upon deformation,

2 $$F={K}_{{{{{{{\mathrm{SF}}}}}}}}\Delta {L}_{{{{{{{\mathrm{SF}}}}}}}}+\eta {{\dot{L}}_{{{{{{{\mathrm{SF}}}}}}}}}={k}_{{{{{{{\mathrm{sf}}}}}}}}{C}_{\alpha }{k}_{{{{{{\mathrm{m}}}}}}}{C}_{{{{{{\mathrm{m}}}}}},{\max }}\left({t}^{\frac{{k}_{\beta ,a}}{{C}_{\alpha }}}+{\left(\frac{{k}_{\beta ,a}}{{C}_{\alpha }}\right)}^{2}{t}^{\left(\frac{{k}_{\beta ,a}}{{C}_{\alpha }}-1\right)}\right)$$

According to our experimental mechanosensation dynamics analysis, after ${t}_{1}$ = 5 min of continuous accumulation of CSK tension, the CSK start to relax which should follow the relaxation of a Kelvin-Voigt material, we can then fit the relaxation process as

3 $$F={k}_{{{{{{{\mathrm{sf}}}}}}}}{C}_{\alpha }{k}_{{{{{{\mathrm{m}}}}}}}{C}_{{{{{{\mathrm{m}}}}}},{\max }}\left({{t}_{1}}^{\frac{{k}_{\beta ,a}}{{C}_{\alpha }}}+{\left(\frac{{k}_{\beta ,a}}{{C}_{\alpha }}\right)}^{2}{{t}_{1}}^{\left(\frac{{k}_{\beta ,a}}{{C}_{\alpha }}-1\right)}\right)\left({e}^{-\left(t-t1\right)\frac{{k}_{{{{{{{\mathrm{rex}}}}}}}}}{\beta }}\right)$$

where ${k}_{{{{{{{\mathrm{rex}}}}}}}}$ is the scaling factor for the exponential relaxation event.

To model the temporal propagation of probability density function (PDF) associated with the instantaneous frequency distribution, We employ Fokker-Planck equation^33^:

$$\frac{\partial p(\omega ,t)}{\partial t}=\frac{\partial (\omega -{\omega }_{c})p(\omega ,t)}{\partial \omega }+D\frac{{\partial }^{2}p(\omega ,t)}{\partial {\omega }^{2}}$$

$${\omega }_{c}=\sqrt{\frac{{K}_{{{{{{{\mathrm{SF}}}}}}}}}{({C}_{\alpha }{V}_{\alpha }{M}_{\alpha }+{C}_{{{{{{\mathrm{m}}}}}}}{V}_{{{{{{\mathrm{m}}}}}}}{M}_{{{{{{\mathrm{m}}}}}}})}-{\left(\frac{\eta }{2\left(\right.{C}_{\alpha }{V}_{\alpha }{M}_{\alpha }+{C}_{{{{{{\mathrm{m}}}}}}}{V}_{{{{{{\mathrm{m}}}}}}}{M}_{{{{{{\mathrm{m}}}}}}}}\right)}^{2}}$$

Where $p(\omega ,t)$ is the temporal PDF of instantaneous angular frequency, ${\omega }_{{{{{{\mathrm{c}}}}}}}=2\pi {f}_{{{{{{\mathrm{c}}}}}}}$ is the central angular frequency that represents the overall spectral response of the VSMC, and $D$ represents the magnitude of environmental noise that diverges the central frequency. In the expression of central angular frequency ${\omega }_{{{{{{\mathrm{c}}}}}}}$, ${V}_{\alpha }$ and ${V}_{{{{{{\mathrm{m}}}}}}}$ are the volume of *α-*actinin2 and myosin solution, while ${M}_{\alpha }$ and ${M}_{{{{{{\mathrm{m}}}}}}}$ are the molecular weights of *α-*actinin2 and myosin molecules. Taking the stationary solution of the above partial differential equation with an error function to account for the skewness of the frequency distribution:

$$p\left(w\right)=\frac{1}{\sqrt{2{pD}{s}_{{{{{{\mathrm{D}}}}}}}}}{e}^{\frac{{(w-{w}_{{{{{{\mathrm{c}}}}}}})}^{2}}{2D{s}_{{{{{{\mathrm{D}}}}}}}}}\left[1+{erf}\left(\left({s}_{{b}_{1}}b+{s}_{{b}_{2}}\right)\frac{w-{w}_{{{{{{\mathrm{c}}}}}}}}{2D{s}_{{{{{{\mathrm{D}}}}}}}}\right)\right]$$

By fitting the modeling PDF curves to the experimental frequency histograms of different cell conditions, we find a good agreement between the theoretical prediction and experimental results, indicating a loss of mechanosensation upon the cellular force generation.

### Calcium imaging

Mice primary VSMC seeded on micropillar array were washed twice using normal physiological saline solution (NPSS) with 140 mM sodium chloride, 5 mM potassium chloride, 2 mM magnesium chloride, 1 mM calcium chloride, 10 mM HEPES, and 10 mM glucose. Cells were then loaded for 20 mins with calcium probe Fluo-4 (F14201, Thermo Fisher Scientific) followed by two washes with NPSS. Imaging was performed using a Zeiss Axio observer with a ×20 objective excited at 480 nm. Before recording data, Ca^2+^ signal was stabilized by imaging VSMC continuously every 1 s for 6 min. To image Ca^2+^ in VSMC upon mechanical stimulation, VSMC were imaged every 200 ms and mechanical stimulation was applied 3 s from the start of the recording. To image Ca^2+^ in VSMC with drug stimulation, Yoda1 (#21904, Cayman Chemical) or recombinant mouse Netrin-1 (#1109-N1, R&D Systems) at a final concentration of 30 µM and 2.5 µg/ml were respectively added to VSMC media. For Piezo1 inhibition assays, cells were preincubated with 2.5 µM GsMTx4 (#STG-100, Alomone labs) for 10 min or 10 µM Dooku1 (#6568, R&D Systems) for 30 min prior to stimulation.

### Statistics and reproducibility

All data were from at least three independent experiments with similar results. Normality test was performed before choosing parametric and non-parametric tests. For experiments with data distribution pass the Shapiro–Wilk test, parametric tests such as two-sided unpaired t-test and ordinary one-way ANOVA test were used for statistical analysis. For those experiments with data distribution does not pass the normality test, non-parametric tests such as two-sided Mann–Whitney test and Kruskal–Wallis test were performed. Analyses were performed with Prism 8 software (GraphPad software Inc., CA, USA). P-value smaller than 0.05 was considered statistically significant and sample sizes and exact *P* values are indicated in all figure legends.

### Reporting summary

Further information on research design is available in the Nature Research Reporting Summary linked to this article.

## Supplementary information

Supplementary Information

Description of Additional Supplementary Files

Supplementary Data 1

Supplementary Data 2

Reporting Summary

Reporting Summary

## Data Availability

Source data are provided with this paper, all RNA sequencing datasets are deposited in Gene Expression Omnibus (GEO)-accession number: GSE186865 for single-cell RNA-sequencing, used in combination with GSE141732^53^ and GSE118237^23^ to generate Figs. 1c–f, 6a, d, e, and 8f, g. GSE152583^26^ was used to generate Supplementary Figs. 1a–c and 6i. Source data underlying graphs are provided in Source Data. All other data supporting the findings of this paper are available from the corresponding authors upon reasonable request. Source data are provided with this paper.

## Source data

Source Data

## Code availability

We used Seurat [https://github.com/satijalab/seurat]^70^ for scRNA-seq analysis. Windowing code for spectral analysis is developed by Danuser’s lab and is available at: https://github.com/DanuserLab/Windowing-Protrusion^32^. MatLab with custom-written codes for modeling VSMC force generation and frequency spectrum analysis is available at: https://github.com/WeiyiQ/Force-generation-frequency-spectrum.
