# Supplementary material for: Microskeletal stiffness promotes aortic aneurysm by sustaining pathological vascular smooth muscle cell mechanosensation via Piezo1
Source: Nat Commun. 2022 Jan 26;13:512. doi: 10.1038/s41467-021-27874-5 (PMC8791986; doi:10.1038/s41467-021-27874-5)
Supplement: Supplementary file 1 — Supplementary Information [file 41467_2021_27874_MOESM1_ESM.pdf]

Supplementary Information for

# Microskeletal stiffness promotes aortic aneurysm by sustaining pathological vascular smooth muscle cell mechanosensation via Piezo1

Weiyi Qian[1†], Tarik Hadi[2†], Michele Silvestro[2], Xiao Ma[3], Cristobal F. Rivera[2], Apratim Bajpai[1], Rui Li[1], Zijing Zhang[1], Hengdong Qu[2], Rayan Sleiman Tellaoui[2], Annanina Corsica[2], Ariadne L. Zias[2], Karan Garg[2], Thomas Maldonado[2], Bhama Ramkhelawon[2,4*], Weiqiang Chen[1,3*]

[1]Department of Mechanical and Aerospace Engineering, New York University, Brooklyn, NY, USA.

[2]Division of Vascular and Endovascular Surgery, Department of Surgery, New York University Langone Medical Center, New York, NY, USA.

[3]Department of Biomedical Engineering, New York University, Brooklyn, NY, USA;

[4]Department of Cell Biology, New York University Langone Medical Center, New York, NY, USA.

† These authors contributed equally to this work

* These authors jointly supervised this work

* Correspondence to: BR (bhama.ramkhelawon@nyulangone.org) and WC (wchen@nyu.edu).

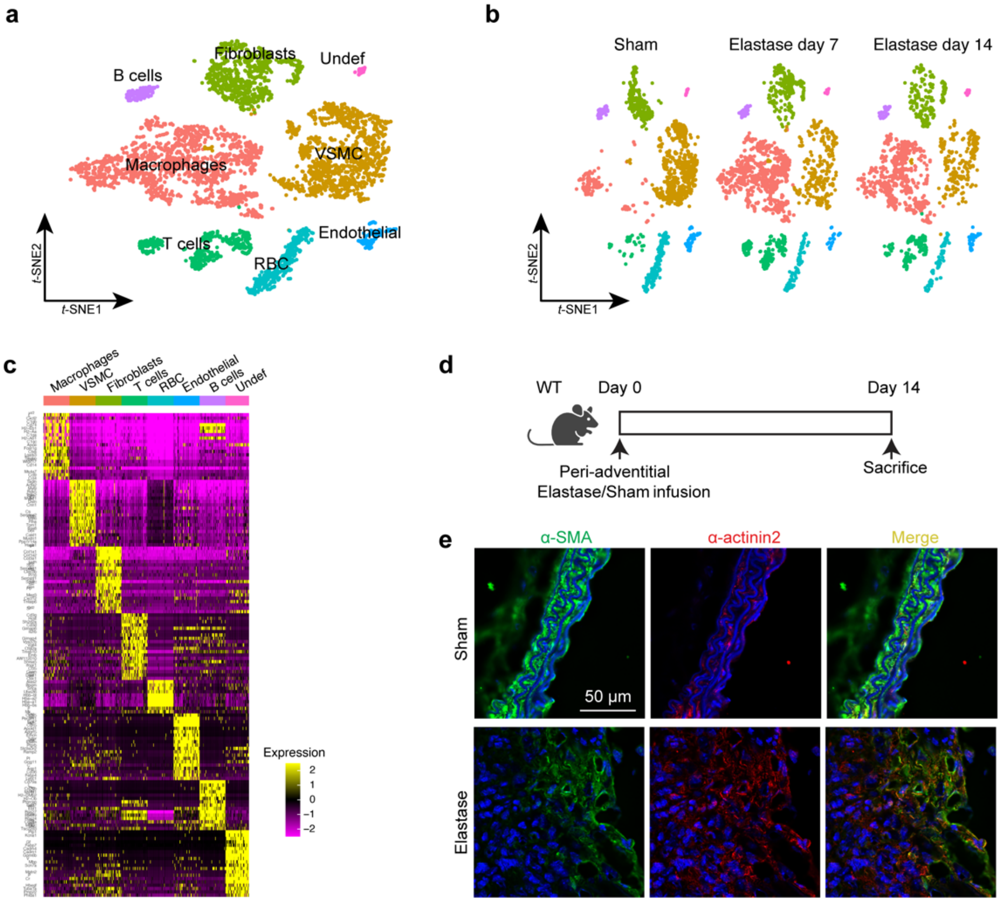


**Supplementary Figure 1.** **(a)** *t*-distributed stochastic neighbor embedding (*t*-SNE) plot of single-cell RNA-sequencing of elastase-induced AAA dataset. Cell clusters annotation is color coded as indicated. (**b**) Individual *t*-SNE plots of sham surgery and elastase-induced AAA dataset at day 7 and day 14 after procedure. Cell clusters are color coded as indicated in (a). **(c)** Heatmap representation of top differentially expressed transcripts across clusters. **(d)** Schematic representation of peri-adventitial elastase infusion and experiment timeline. **(e)** Immunofluorescence staining of α-actinin2 (red) and α-SMA (green) in aortas of mice infused with elastase or sham. *n* = 4 mice per group. Nuclei are stained with DAPI (blue). Mouse icon in d is created with BioRender.com.

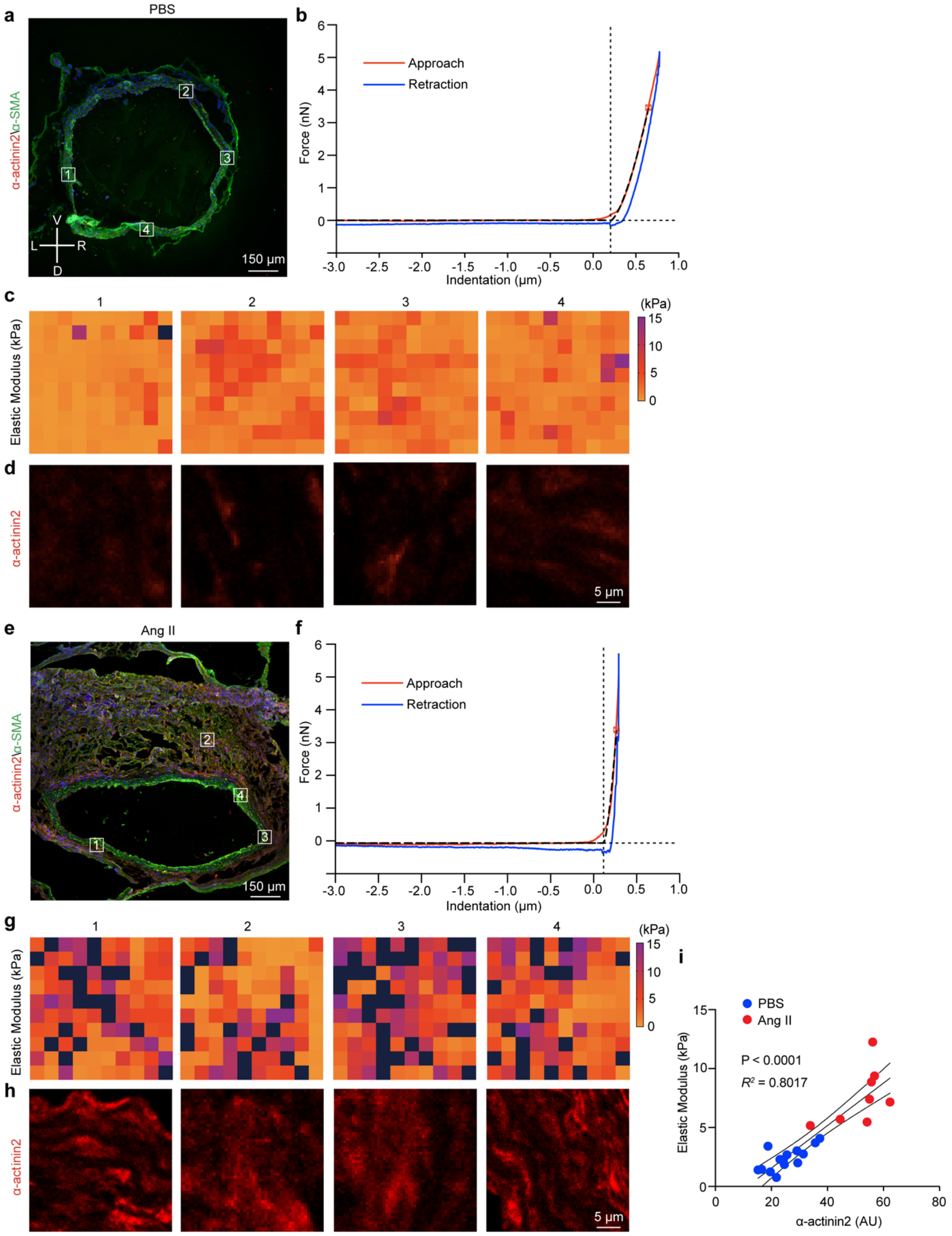


**Supplementary Figure 2.** (**a&e**) Immunofluorescence staining of α-actinin2 (red) α-SMA (green) in aortas of mice perfused with PBS **(a)** or Ang II **(e)**. *n* = 3 aortic sections per group. Nuclei are stained with DAPI (blue). Numbered squares highlight regions of elastic modulus measurements. (**b&f**) Representative force curves showing the approach (red) and retraction (blue) curves for one AFM measurement of aortic samples of mice perfused with PBS **(**b**)** or Ang II **(f)**. Cross-hairs indicate contact point position and force. Red square with cross on the approach curves indicates the definite force to fit the curve. The fit to the baseline and the Hertz model is indicated by a

dotted black line. Heatmap representation of local elastic modulus **(c, g)** and corresponding immunofluorescence staining of α-actinin2 **(d, h)** from numbered regions in (a) and (e). (**i**) Correlation between elastic modulus and α-actinin2 staining intensity in ApoE$^{-/-}$ mice perfused with PBS (blue dots) or Ang II (red dots). AU: Arbitrary Unit. Data is presented as linear regression showing 95% confidence bands. $P < 0.0001$. *n* = 15 square regions for PBS and 8 for Ang II from 3 aortic sections per group. Source data are provided as a Source Data file.

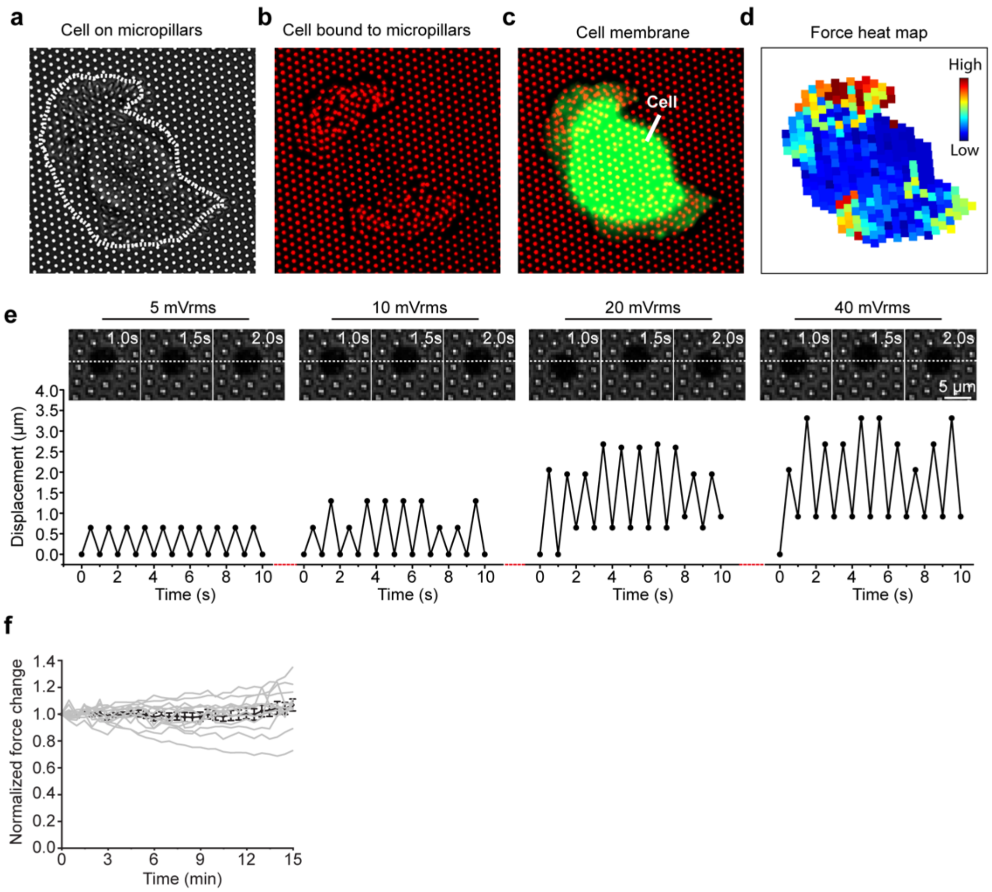


**Supplementary Figure 3. (a-c)** Representative images showing a VSMC seeded on micropillar array (a) and the bent micropillar array functionalized with fibronectin and Alexa Fluor™ 647 Conjugate fibrinogen (b). VSMC membrane is labelled with CellTracker™ Green CMFDA fluorescent dye and merged with the underlying micropillar (c). (**d**) Representative heat map showing the CSK tension of the VSMC in (a) by quantifying the displacements of bent micropillar from their unbent positions. (**e**) Time-lapse brightfield images and quantified temporal translational displacement of MB during the 10 sec US stimulation with different applied power. (**f**) Normalized force change of VSMC without mechanical stimulation from ultrasound tweezer. *n* = 9 cells. Data are represented as mean values ± SEM (black) and each individual value (grey). Source data are provided as a Source Data file.

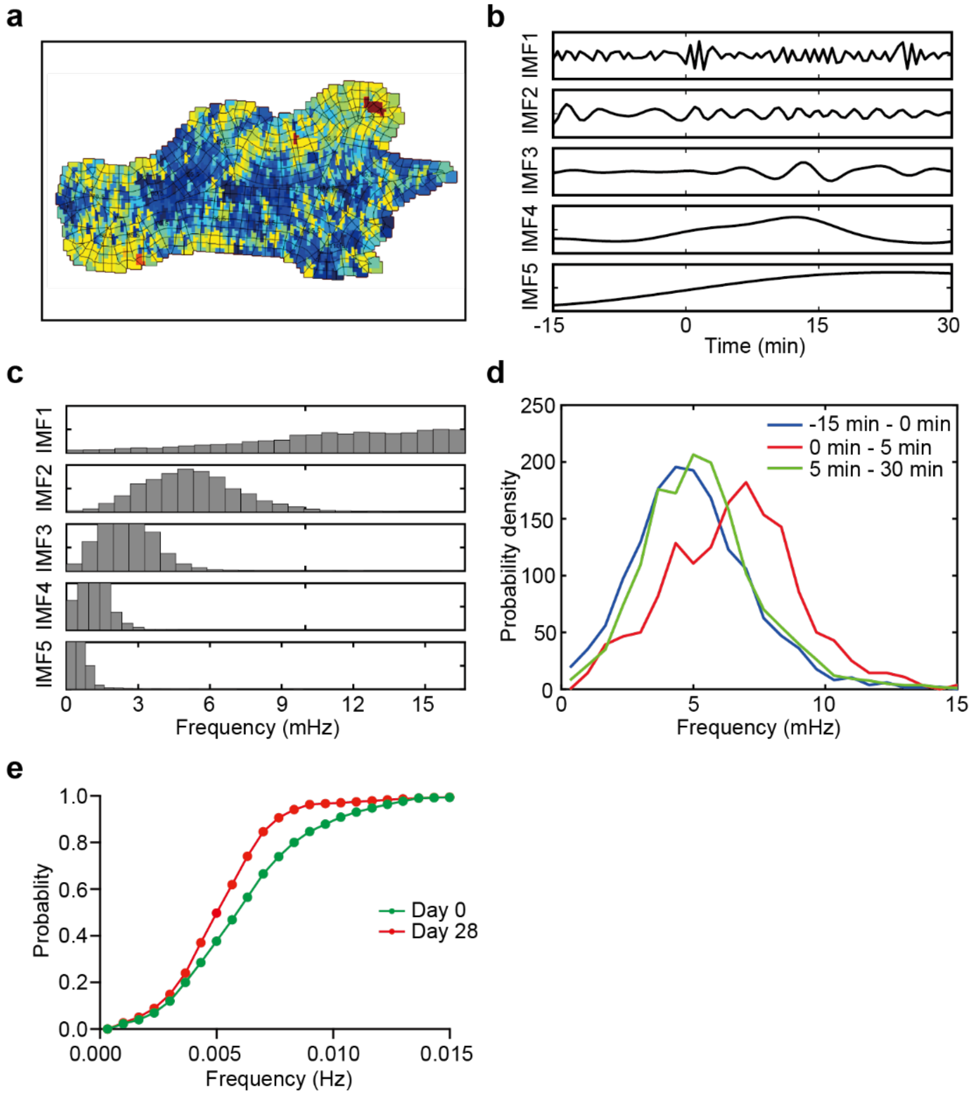


**Supplementary Figure 4**. (**a**) Overlaid sampling window upon a healthy mice VSMC. (**b**) Empirical mode decomposition (EMD) of the example force dynamics time series. (**c**) Instantaneous frequency distributions of each IMF for the whole cell, calculated by the Hilbert transform. (**d**) Frequency shift in response to the mechanical activation during 0-5min, compared with the non-activation phase (-15min-0min) and recovery phase (5min-30min). (**e**) Cumulative density functions (CDFs) of frequency distribution for PBS and Ang II treated mice. $P$ = 0.010364 based on Kolmogorov–Smirnov (KS) test. Source data are provided as a Source Data file.

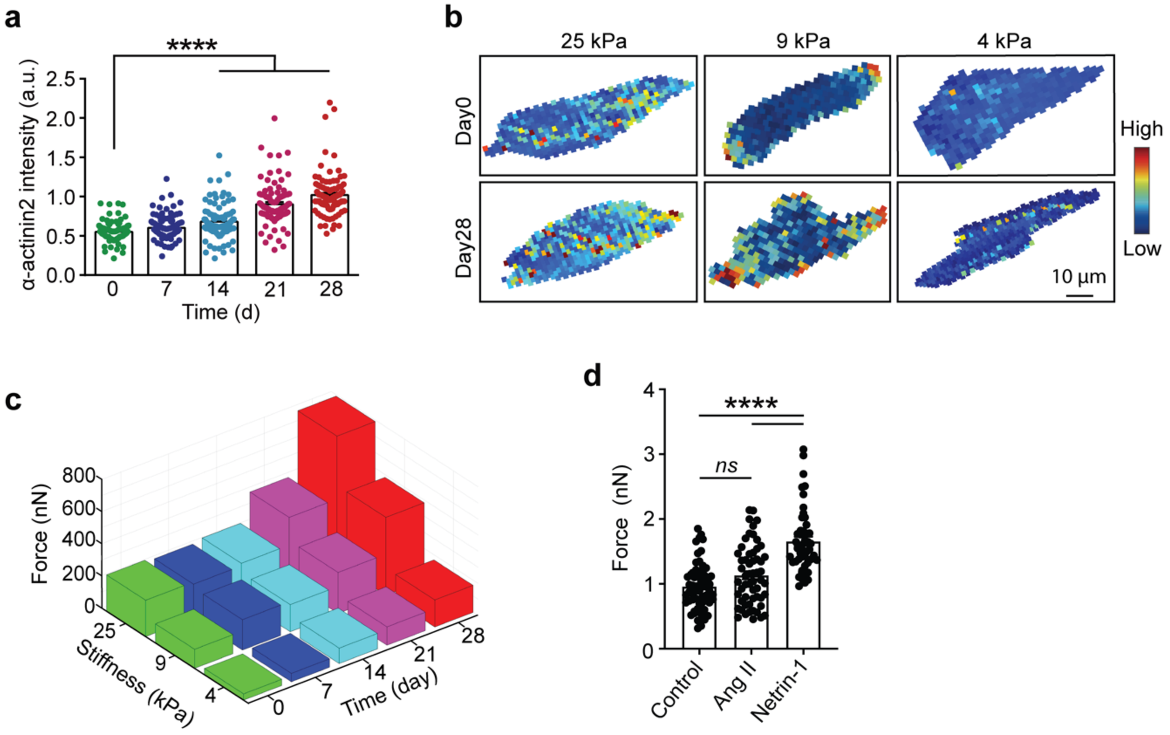


**Supplementary Figure 5. (a)** Quantification of α-actinin2 intensity in VSMC isolated from aorta of mice perfused with Ang II for 0 (control), 7, 14, 21, or 28 days. *n* = 99 cells per group. One-way ANOVA followed by Dunn's multiple comparisons test. ****P < 0.0001. Data are presented as mean values ± SEM. **(b)** Representative heat maps showing the measured traction force of VSMCs isolated from control mice and mice treated with Ang II for 28 days using micropillar array with different stiffness as indicated. Scale bar: 10 μm. **(c)** 3D bar showing quantified mean total force of VSMCs isolated from mice treated with Ang II for different days using micropillar array of different stiffness as indicated. *n* = 20 cells per condition. (**d**) Measured VSMC baseline contractility under the treatment of PBS (control), Ang II, and Netrin-1for 24 hours. *n* = 60, 57, and 49 cells for Control, Ang II, and Netrin-1, respectively. Kruskal-Wallis test followed by Dunn's multiple comparisons test. *ns*: no significant difference and ****P < 0.0001. Source data are provided as a Source Data file.

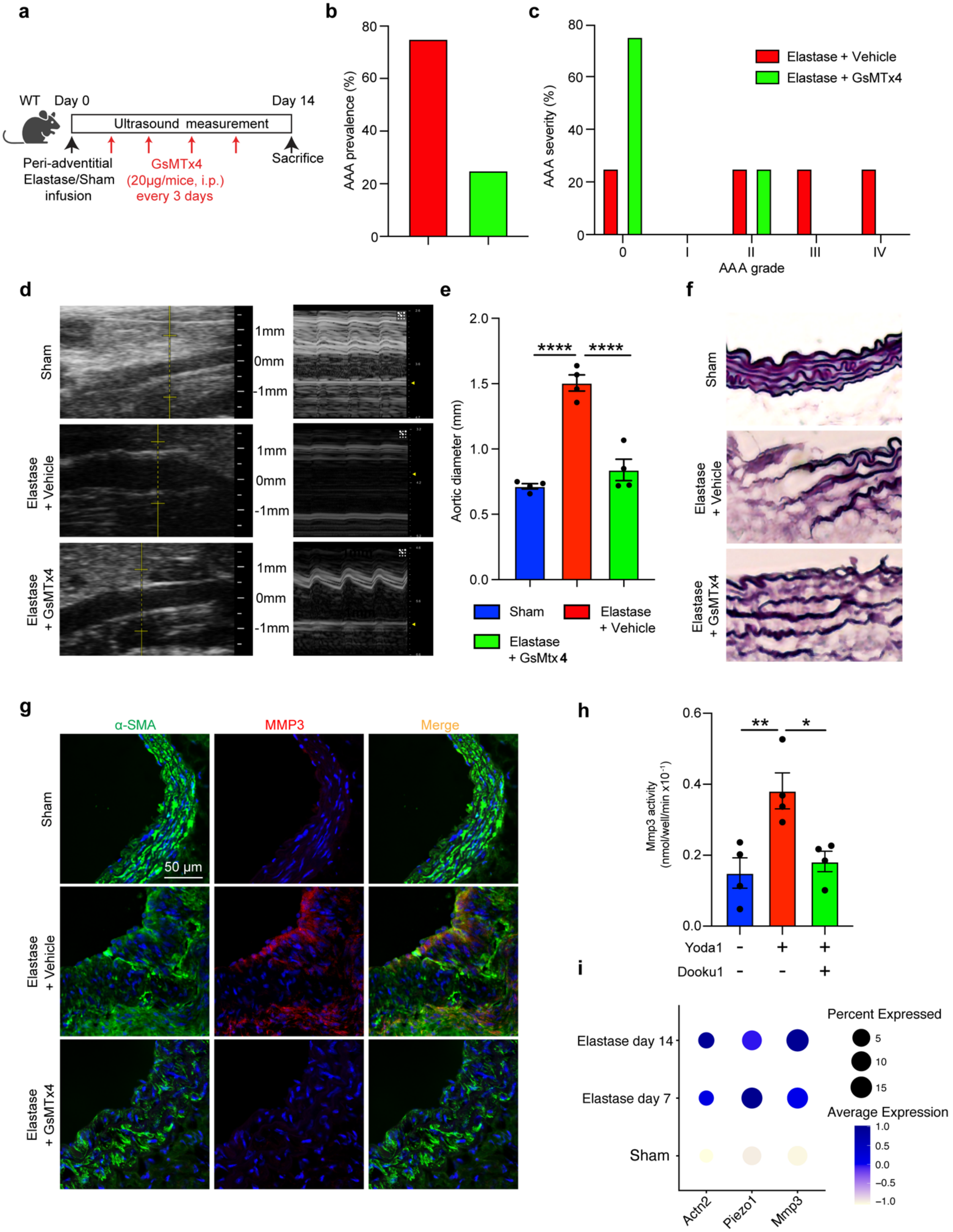


**Supplementary Figure 6**. (**a**) Schematic representation of GsMTx4 treatment during AAA induction using the peri-adventitial elastase infusion model. *i.p.*, intraperitoneal. AAA prevalence **(b),** AAA severity score **(c)** in mice perfused with elastase with vehicle or GsMTx4. **(d)** Representative color Doppler ultrasound images and **(e)** measurements of maximum transverse aortic diameter at day 14. *n* = 4 mice per group. One-way ANOVA followed by Turkey's multiple comparisons test. ****P < 0.0001. Data are presented as mean values ± SEM. **(f)** Verhoeff-Van

Gieson elastin staining in aortas of mice infused with elastase and treated with GsMTx4 or vehicle. *n* = 4 mice per group. (**g**) Immunofluorescence staining of Mmp3 (red) and α-SMA (green) in aortas of mice perfused with elastase with vehicle or GsMTx4. *n* = 4 mice per group. Nuclei are stained with DAPI (blue). **(h)** Mmp3 activity assay in VSMC stimulated with Yoda1 or control, with or without Dooku1. *n* = 4 mice per group. One-way ANOVA followed by Turkey's multiple comparisons test. *P = 0.0203 and **P = 0.0088. Data are presented as mean values ± SEM. **(i)** Dotplot representation of *Actn2*, *Piezo1* and *Mmp3* in vascular smooth muscle cells cluster in single-cell RNA-sequencing of elastase-induced AAA dataset, across conditions as indicated. Mouse icon in a is created with BioRender.com. Source data are provided as a Source Data file.

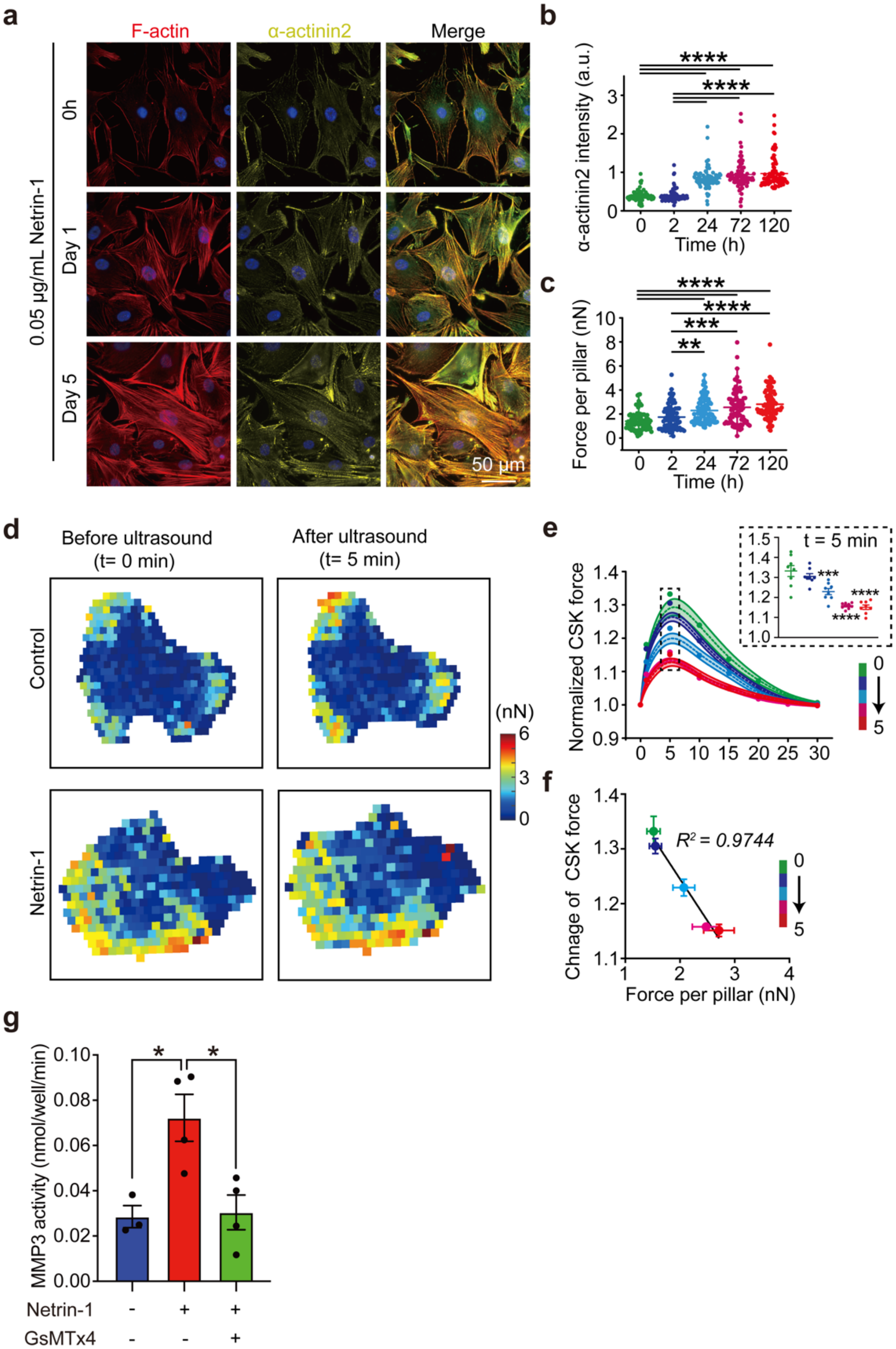


**Supplementary Figure 7**. (**a-c**) Representative IF images of α-actinin2 (Yellow) and F-actin (red) (a), quantification of α α-actinin2 signal intensity (b), and force per pillar measurements (c) in human VSMC stimulated with Netrin-1 as indicated. For (b), *n* = 86 cells per group. Kruskal-Wallis test followed by Dunn's multiple comparisons test. ****P < 0.0001. Data are presented as mean values ± SEM. For (c), *n* = 86 cells per group. Kruskal-Wallis test followed by Dunn's multiple comparisons test. **P = 0.0059, ***P = 0.0005, and ****P < 0.0001. Data are presented as mean values ± SEM. **(d)** Representative heatmap representation of baseline micropillar traction force or in response to ultrasound stimulation (5 and 30 minutes after the stimulation) of single human VSMC stimulated without/with Netrin-1 as indicated. **(e)** Temporal profiles of normalized CSK

tension per pillar of human VSMC treated with Netrin-1 (color coded as in b&c) in response to 10-sec mechanical stress. Color-coded dash lines represent mean value and color-coded area represent connected SEM. Enlarged dashed box shows the normalized CSK force at t = 5 min for each group. *n* = 8 cells per group. One-way ANOVA followed by Turkey's multiple comparisons test. ***P = 0.0007 and ****P < 0.0001. Data are presented as mean values ± SEM. **(f)** Correlation analysis of human VSMC progressive basal CSK tension in AAA and maximum force generation in instantaneous mechanosensation. *n* = 8 cells per group. Data are presented as mean values ± SEM. **(g)** Mmp3 activity assay in human VSMC stimulated with Netrin-1 or control, with or without GsMTx4. *n* = 3, 4, and 4 independent experiments for control, Netrin-1, and Netrin-1 + GsMTx4, respectively. One-way ANOVA followed by Turkey's multiple comparisons test. *P = 0.0199 (control *vs* Netrin-1) and *P = 0.0169 (Netrin-1 *vs* Netrin-1 + GsMTx4). Data are presented as mean values ± SEM. Source data are provided as a Source Data file.

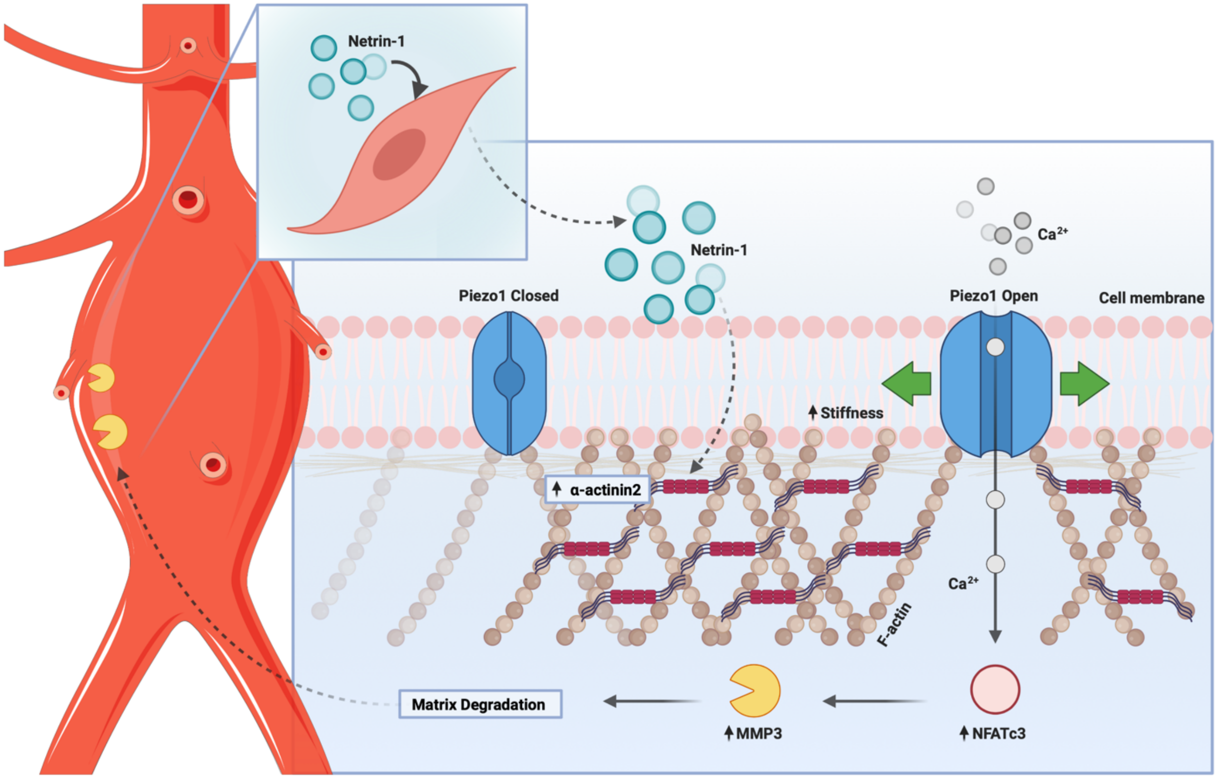


**Supplementary Figure 8**. Schematic representation of our findings. The graphic is created with BioRender.com.

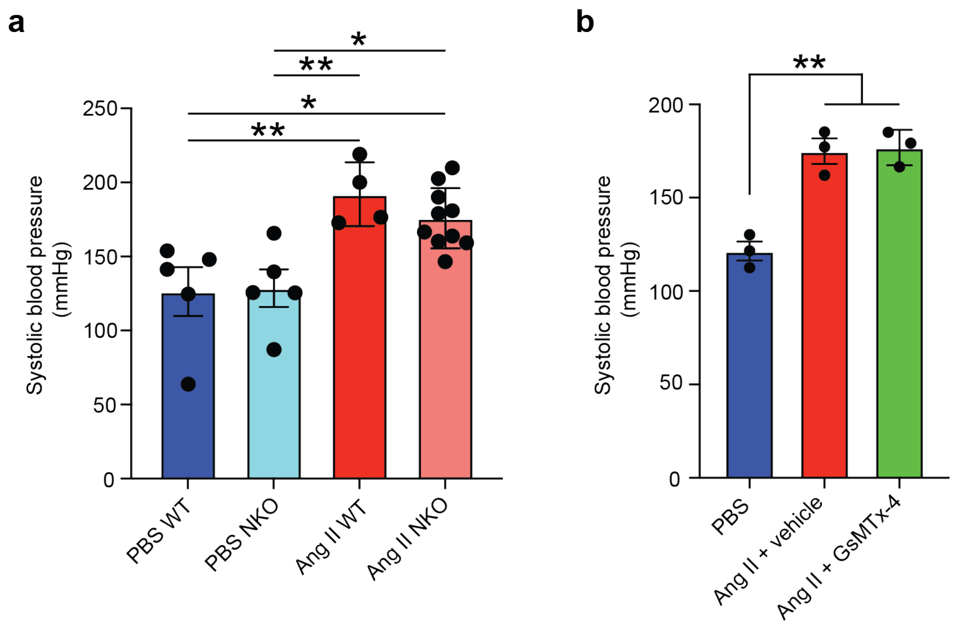


**Supplementary Figure 9. (a)** Systolic blood pressure in WT or NKO mice perfused with PBS or Ang II. *n* = 5, 5, 4, and 10 mice for PBS WT, PBS NKO, Ang II WT, and Ang II NKO, respectively. One-way ANOVA followed by Turkey's multiple comparisons test. *P = 0.0122 (PBS WT *vs* Ang II NKO), *P = 0.0176 (PBS NKO *vs* Ang II NKO), **P = 0.0065 (PBS WT *vs* Ang II WT), and **P = 0.0088 (PBS NKO *vs* Ang II WT). Data are presented as mean values ± SEM. **(b)** Systolic blood pressure in ApoE$^{-/-}$ mice perfused with PBS or Ang II and treated with GsMTx4 or vehicle. *n* = 3 mice per group. One-way ANOVA followed by Turkey's multiple comparisons test. **P = 0.0016 (PBS *vs* Ang II + vehicle) and **P = 0.0013 (PBS *vs* Ang II +GsMTx4). Data are presented as mean values ± SEM. Source data are provided as a Source Data file.

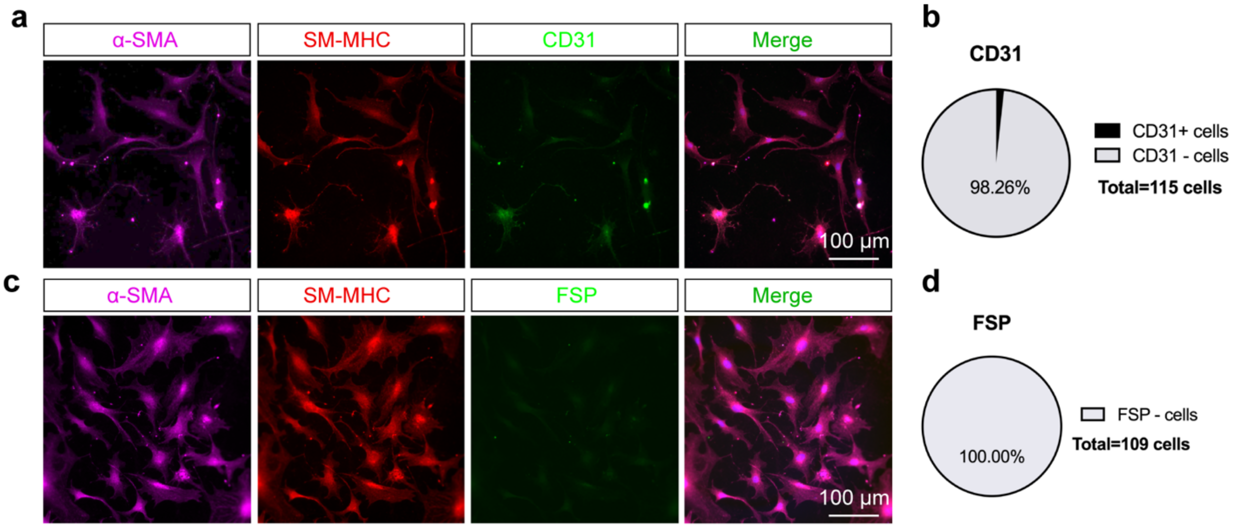


**Supplementary Figure 10. (a-b),** Representative immunofluorescent images of isolated VSMC stained for α-SMA, SM-MHC, and CD31(a) and quantification (b). *n* = 115 cells. **(c-d),** Representative immunofluorescent images of isolated VSMC stained for α-SMA, SM-MHC, and FSP (c) and quantification showing all isolated VSMC are FSP negative (d). *n* = 109 cells. Source data are provided as a Source Data file.

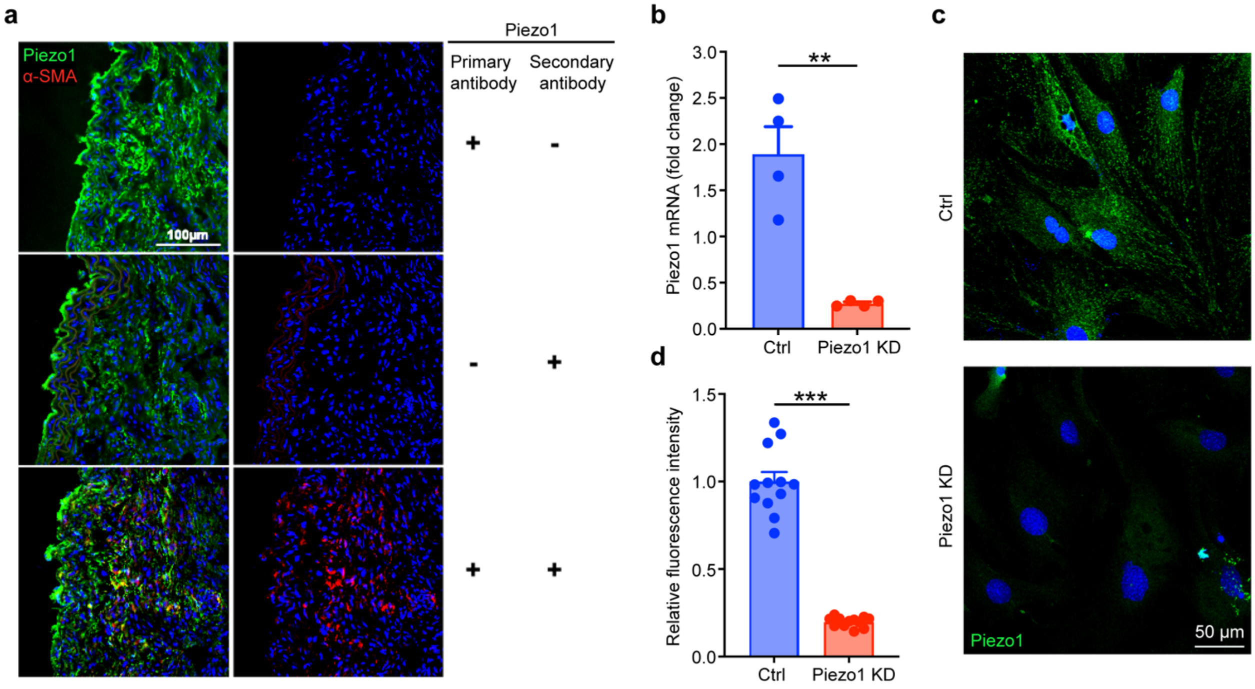


**Supplementary Figure 11**. **Validation of anti-Piezo1 antibody**. **(a)** Immunofluorescence staining of α-SMA (green) and Piezo1 (red) in serial sections of aortas of mice exposed to Ang II. Primary and secondary antibody for Piezo1 detection were added as indicated. Nuclei are stained with DAPI (blue). **(b)** Quantitative-RT-PCR (qPCR) analysis of *Piezo1* mRNA levels in mouse VSMCs treated with non-targeting ('Ctrl') or Piezo1 siRNA (''Piezo1 KD''). n = 4 independent experiments per group. Two-sided unpaired *t*-test. **P = 0.0016. Data are presented as mean values ± SEM. **(c-d)** Representative IF images (c) and quantification of relative IF intensity (d) of Piezo1staining in mouse VSMCs treated with non-targeting or Piezo1 siRNA. n = 12 images from 4 independent experiments per group. Two-sided unpaired *t*-test. ****P < 0.0001. Data are presented as mean values ± SEM. Source data are provided as a Source Data file.

**Supplementary Table 1.** List of modelling parameter

| Sym. | Definition | *Health* | *Day 7* | *Day 14* | *Day 21* | *Day 28* |
|---|---|---|---|---|---|---|
| $k_{sf}$ | Scaling constant for actin stress fiber (nN/μm/μM) | 16.6 | – | – | – | – |
| $C_{\alpha}$ | Concentration of α-actinin2 (μM) | 14.5 | 16 | 16.8 | 18 | 30 |
| $k_m$ | Scaling constant for activated myosin (μm/μM) | $3.6 \times 10^{-5}$ | – | – | – | – |
| $C_{m,max}$ | Maximum activated myosin (μM) | 1.25 | – | – | – | – |
| $k_{\beta,\alpha}$ | Scaling constant for CSK viscoelasticity | 8 | – | – | – | – |
| $k_{rex}$ | Scaling constant for CSK relaxation | 1200 | – | – | – | – |
| $\beta$ | Viscoelasticity index | 0.55 | 0.5 | 0.48 | 0.44 | 0.27 |
