# Supplementary material for: Microskeletal stiffness promotes aortic aneurysm by sustaining pathological vascular smooth muscle cell mechanosensation via Piezo1
Source: Nat Commun. 2022 Jan 26;13:512. doi: 10.1038/s41467-021-27874-5 (PMC8791986; doi:10.1038/s41467-021-27874-5)
Supplement: Supplementary file 2 — Description of Additional Supplementary Files [file 41467_2021_27874_MOESM2_ESM.pdf]

Description of Additional Supplementary Files

File Name: Supplementary Data 1
Description: Transcriptome with differential expression patterns in VSMC niched in AAA compared to controls mice.

File Name: Supplementary Data 2
Description: Library of differentially expressed genes from published RNAseq dataset of aorta isolated from Ntn1$^{flox/flox}$ mice and Ntn1$^{flox/flox}$LysMcre$^{+/-}$ mice.
