# Supplementary material for: Microskeletal stiffness promotes aortic aneurysm by sustaining pathological vascular smooth muscle cell mechanosensation via Piezo1
Source: Nat Commun. 2022 Jan 26;13:512. doi: 10.1038/s41467-021-27874-5 (PMC8791986; doi:10.1038/s41467-021-27874-5)
Supplement: Supplementary file 6 — Reporting Summary [file 41467_2021_27874_MOESM6_ESM.pdf]

nature research

Corresponding author(s): Bhama Ramkhelawon, Weiqiang Chen

Last updated by author(s): May 1, 2021

Reporting Summary

Nature Research wishes to improve the reproducibility of the work that we publish. This form provides structure for consistency and transparency in reporting. For further information on Nature Research policies, see our Editorial Policies and the Editorial Policy Checklist.

Statistics

For all statistical analyses, confirm that the following items are present in the figure legend, table legend, main text, or Methods section.

n/a	Confirmed	
[ ]	[x]	The exact sample size (n) for each experimental group/condition, given as a discrete number and unit of measurement
[ ]	[x]	A statement on whether measurements were taken from distinct samples or whether the same sample was measured repeatedly
[ ]	[x]	The statistical test(s) used AND whether they are one- or two-sided<br>*Only common tests should be described solely by name; describe more complex techniques in the Methods section.*
[x]	[ ]	A description of all covariates tested
[ ]	[x]	A description of any assumptions or corrections, such as tests of normality and adjustment for multiple comparisons
[ ]	[x]	A full description of the statistical parameters including central tendency (e.g. means) or other basic estimates (e.g. regression coefficient) AND variation (e.g. standard deviation) or associated estimates of uncertainty (e.g. confidence intervals)
[ ]	[x]	For null hypothesis testing, the test statistic (e.g. F, t, r) with confidence intervals, effect sizes, degrees of freedom and P value noted<br>*Give P values as exact values whenever suitable.*
[x]	[ ]	For Bayesian analysis, information on the choice of priors and Markov chain Monte Carlo settings
[x]	[ ]	For hierarchical and complex designs, identification of the appropriate level for tests and full reporting of outcomes
[ ]	[x]	Estimates of effect sizes (e.g. Cohen's d, Pearson's r), indicating how they were calculated

Our web collection on statistics for biologists contains articles on many of the points above.

Software and code

Policy information about availability of computer code

Data collection	Immunofluorescence images were acquired using the Zeiss Efficient Navigation (ZEN) software (Carl Zeiss); Aortic diameter and Pulse Wave Velocity (PWV) was assessed using a Vevo 2100 ultrasound imaging platform (FUJIFILM VisualSonics); MFP-3D-BIO Atomic Force Microscope (Asylum Research, Oxford Instruments, Santa Barbara, CA) was used for AFM measurement.
Data analysis	Cell contractility was analyzed by the Cellogram algorithm from Ref. 66 and previously developed MATLAB programs (Mathworks) from Ref. 64. Spectral analysis of the cell force dynamics was which was analyzed by a previously developed algorithm from Ref. 33. The Asylum Research Software was used for the AFM-based stiffness analysis. Learning Unsupervised Means of Spectra (LUMoS) method (ImageJ) was used for analysis of mmunofluorescence staining intensity of some markers of cell. RNA sequencing dataset of WT and NKO previously published by our group was re-analyzed by using the DESEQ2 package from Bioconductor running on R statistical program. Graphpad Prism 8.1.1 was used for plotting and statistic analysis. Adobe Illustrator was used for organizing figures.

For manuscripts utilizing custom algorithms or software that are central to the research but not yet described in published literature, software must be made available to editors and reviewers. We strongly encourage code deposition in a community repository (e.g. GitHub). See the Nature Research guidelines for submitting code & software for further information.

Data

Policy information about availability of data

All manuscripts must include a data availability statement. This statement should provide the following information, where applicable:

- Accession codes, unique identifiers, or web links for publicly available datasets
- A list of figures that have associated raw data
- A description of any restrictions on data availability

All data associated with this study are present in the paper and Supplementary Information. Raw data are available from the corresponding author on reasonable

request.

Field-specific reporting

Please select the one below that is the best fit for your research. If you are not sure, read the appropriate sections before making your selection.

☒ Life sciences ☐ Behavioural & social sciences ☐ Ecological, evolutionary & environmental sciences

For a reference copy of the document with all sections, see nature.com/documents/nr-reporting-summary-flat.pdf

Life sciences study design

All studies must disclose on these points even when the disclosure is negative.

Sample size	No predetermination of sample size was conducted by statistical method.
Data exclusions	No data were excluded.
Replication	All data were from at least three independent experiments, and presented as means ± s.e.m.
Randomization	In each experimental group, mice/samples were randomly distributed.
Blinding	Investigators were blind to PWV analysis after measurements.

Reporting for specific materials, systems and methods

We require information from authors about some types of materials, experimental systems and methods used in many studies. Here, indicate whether each material, system or method listed is relevant to your study. If you are not sure if a list item applies to your research, read the appropriate section before selecting a response.

Materials & experimental systems

n/a	Involved in the study	
☐	☒	Antibodies
☐	☒	Eukaryotic cell lines
☒	☐	Palaeontology and archaeology
☐	☒	Animals and other organisms
☐	☒	Human research participants
☒	☐	Clinical data
☒	☐	Dual use research of concern

Methods

n/a	Involved in the study	
☒	☐	ChIP-seq
☒	☐	Flow cytometry
☒	☐	MRI-based neuroimaging

Antibodies

Antibodies used	Primary antibodies: anti-α-actinin2 (#14221-1-AP, Proteintech), anti-Piezo1, (#15939-1-AP, Proteintech), anti-alpha-actin (sc-130656, Santa Cruz Biotechnology), anti-alpha-smooth muscle actin (#48938S, Cell Signaling). Alexa Fluor 488 and 647 conjugated anti-IgG antibodies (Invitrogen) were used for fluorescent signal detection in immunofluorescence staining. Alexa Fluor 555 conjugated to phalloidin (Invitrogen) and 4',6-diamidino-2-phenylindole (DAPI; Invitrogen) were used for visualization of actin microfilaments and nucleus respectively. HRP conjugated secondary antibodies, goat anti-rabbit (7074P2, Cell Signaling) or goat anti-mouse (A9917, Sigma Aldrich) were used in Western blotting study.
Validation	Validation of the primary anti-Piezo1 antibody were performed to confirm the specificity. All primary antibodies have been referenced in previous publications per manufacture's website.

Eukaryotic cell lines

Policy information about cell lines

Cell line source(s)	Human aorta vascular smooth muscle cell (Lonza).
Authentication	The human aorta vascular smooth muscle cell were stained positive for alpha smooth muscle actin and negative for von. Willebrand Factor VIII
Mycoplasma contamination	All cells test negative for mycoplasma.
Commonly misidentified lines (See ICLAC register)	None of the cells used is in ICLAC.

Animals and other organisms

Policy information about studies involving animals; ARRIVE guidelines recommended for reporting animal research

Laboratory animals	8-week old male C57BL/6J (WT), LysMcre and ApoE−/− mice were used and bought from Jackson Laboratories
Wild animals	The study did not involve wild animals
Field-collected samples	No field-collected samples were involved in this study.
Ethics oversight	The US Department of Agriculture Animal Welfare Act, the Public Health Service Policy for the Humane Care and Use of Laboratory Animals and the New York University School of Medicine's Institutional Animal Care and Use Committee approved all experimental procedures which were conducted according to their guidelines.

Note that full information on the approval of the study protocol must also be provided in the manuscript.

Human research participants

Policy information about studies involving human research participants

Population characteristics	Patients were not discriminated according to age, gender, genotype information or past/current diagnosis
Recruitment	Aneurysmal tissue was collected from individuals undergoing open aortic aneurysm repair. Informed consent was obtained for each subject. The study was authorized with IRB approval number i16-01807. Healthy cadaver tissues from multi-organ donors who had been confirmed as brain-dead were provided by LiveOnNY organization (NY, New York). Informed consent for the use of the samples was obtained from the donors or their families.
Ethics oversight	All studies were conducted in accordance with policies set forth by the NYU Institutional Review Board (IRB).

Note that full information on the approval of the study protocol must also be provided in the manuscript.
